# LRRTM3 Interacts with APP and BACE1 and Has Variants Associating with Late-Onset Alzheimer’s Disease (LOAD)

**DOI:** 10.1371/journal.pone.0064164

**Published:** 2013-06-04

**Authors:** Sarah Lincoln, Mariet Allen, Claire L. Cox, Louise P. Walker, Kimberly Malphrus, Yushi Qiu, Thuy Nguyen, Christopher Rowley, Naomi Kouri, Julia Crook, V. Shane Pankratz, Samuel Younkin, Linda Younkin, Minerva Carrasquillo, Fanggeng Zou, Samer O. Abdul-Hay, Wolfdieter Springer, Sigrid B. Sando, Jan O. Aasly, Maria Barcikowska, Zbigniew K. Wszolek, Jada M. Lewis, Dennis Dickson, Neill R. Graff-Radford, Ronald C. Petersen, Elizabeth Eckman, Steven G. Younkin, Nilüfer Ertekin-Taner

**Affiliations:** 1 Mayo Clinic Florida, Department of Neuroscience, Jacksonville, Florida, United States of America; 2 Mayo Clinic Florida, Biostatistics Unit, Jacksonville, Florida, United States of America; 3 Mayo Clinic Minnesota, Department of Biostatistics, Rochester, Minnesota, United States of America; 4 Department of Neurology, St. Olav’s Hospital, Trondheim, Norway; 5 Department of Neuroscience, Norwegian University of Science and Technology, NTNU, Trondheim, Norway; 6 Department of Neurodegenerative Disorders, Medical Research Centre, Polish Academy of Sciences, Warsaw, Poland; 7 Mayo Clinic Florida, Department of Neurology, Jacksonville, Florida, United States of America; 8 University of Florida, Department of Neuroscience, Gainesville, Florida, United States of America; 9 Mayo Clinic Minnesota, Department of Neurology, Rochester, Minnesota, United States of America; University of Kentucky, United States of America

## Abstract

Leucine rich repeat transmembrane protein 3 (LRRTM3) is member of a synaptic protein family. *LRRTM3* is a nested gene within α-T catenin (*CTNNA3*) and resides at the linkage peak for late-onset Alzheimer’s disease (LOAD) risk and plasma amyloid β (Aβ) levels. *In-vitro* knock-down of *LRRTM3* was previously shown to decrease secreted Aβ, although the mechanism of this is unclear. In SH-SY5Y cells overexpressing APP and transiently transfected with LRRTM3 alone or with BACE1, we showed that LRRTM3 co-localizes with both APP and BACE1 in early endosomes, where BACE1 processing of APP occurs. Additionally, LRRTM3 co-localizes with APP in primary neuronal cultures from Tg2576 mice transduced with LRRTM3-expressing adeno-associated virus. Moreover, LRRTM3 co-immunoprecipitates with both endogenous APP and overexpressed BACE1, in HEK293T cells transfected with LRRTM3. SH-SY5Y cells with knock-down of *LRRTM3* had lower *BACE1* and higher *CTNNA3* mRNA levels, but no change in *APP*. Brain mRNA levels of *LRRTM3* showed significant correlations with *BACE1*, *CTNNA3* and *APP* in ∼400 humans, but not in *LRRTM3* knock-out mice. Finally, we assessed 69 single nucleotide polymorphisms (SNPs) within and flanking *LRRTM3* in 1,567 LOADs and 2,082 controls and identified 8 SNPs within a linkage disequilibrium block encompassing 5′UTR-Intron 1 of *LRRTM3* that formed multilocus genotypes (MLG) with suggestive global association with LOAD risk (p = 0.06), and significant individual MLGs. These 8 SNPs were genotyped in an independent series (1,258 LOADs and 718 controls) and had significant global and individual MLG associations in the combined dataset (p = 0.02–0.05). Collectively, these results suggest that protein interactions between LRRTM3, APP and BACE1, as well as complex associations between mRNA levels of *LRRTM3, CTNNA3, APP* and *BACE1* in humans might influence APP metabolism and ultimately risk of AD.

## Introduction

LRRTM3 is a member of the synaptic leucine rich repeat transmembrane family with four highly conserved members, each with distinct brain distributions [1]. Three LRRTMs (*LRRTM1-3*) reside within introns of an α-catenin gene and are transcribed in the direction opposite to that of their α-catenin partners [1]. *LRRTM3* exists within the seventh intron of *CTNNA3* (also known as *VR22*), encoding α-T-catenin. Both *LRRTM3* and *CTNNA3* are located at the genetic linkage peaks for plasma levels of amyloid ß (Aß) identified by our group in extended late-onset Alzheimer’s disease (LOAD) families [2] and for LOAD risk detected in an independent sib-pair study [3], making both genes interesting positional candidate LOAD risk genes.

*LRRTM3* is also a functionally relevant candidate LOAD gene, as it is structurally similar to a family of neuronal receptors that includes the NOGO receptor, which inhibits neuronal regeneration and APP processing [4]. In a high-throughput siRNA screening of 15,200 genes by Majercak et al. [4], siRNA targeting of *LRRTM3* inhibited the secretion of Aβ40, Aβ42, and sAPPβ in HEK293 and SH-SY5Y cells overexpressing amyloid precursor protein (APP), but had no effect on sAPPα or γ-secretase cleavage of the 99 amino acid C-terminal fragment of APP. Given these results and the lack of an effect of LRRTM3 knock-down on total BACE activity, mRNA levels of APP processing secretases, or APP levels in that study, the authors concluded that LRRTM3 mediates BACE1 processing of APP potentially by vesicle trafficking or signaling. Despite additional scant evidence for promotion of APP processing by LRRTM3 [5], [6], we are not aware of any studies to date that investigate the interaction between APP, BACE1 and LRRTM3.

There have been several genetic association studies on the chromosome 10q linkage region harboring *CTNNA3* and *LRRTM3*. Our group first published a fine mapping study of this region that investigated the effect of 51 SNPs on the intermediate Aβ phenotype; and identified two intronic SNPs (rs7070570, rs12357560) in tight linkage disequilibrium (LD) that accounted for the chromosome 10q Aβ linkage signal in our extended families [2], replication of the Aβ association in a second independent set of families [7] and decay of this association signal within the large *CTNNA3* genic region. These findings strongly implicated *CTNNA3* as a LOAD gene that affects Aβ42. Subsequently, there have been numerous reports of both positive [8], [9], [10], [11] and negative [12], [13] association between *CTNNA3* SNPs and AD risk, highlighting the complexity of this region [14].

*LRRTM3* genetic associations with AD risk was first studied, in a follow-up to our report on *CTNNA3* by Martin et al., who assessed 11 variants in both *CTNNA3* and its nested gene *LRRTM3* and identified evidence of association with AD for both genes [8]. In a two-stage analysis assessing for heterogeneity and gene-gene interactions in 22 candidate genes, *LRRTM3* SNPs were identified to be the most influential in determining clusters for AD risk [15]. Furthermore in this study, *LRRTM3* SNP multilocus genotypes (MLGs) were shown to have statistical interactions with *PLAU*, previously implicated in AD risk and Aβ levels [16], as well as *CDC2* and *ACE* in conferring AD risk. Additional studies identified multilocus genotypes composed of SNPs in *ACE*, *LRRTM3* and *A2M* with significant AD risk association, where all three genes have implications in Aβ metabolism [17]. Finally, a study in Caucasian-American and Carribean-Hispanic case-control series, assessed 5 SNPs and identified SNP and haplotype associations with AD risk, in especially the latter group [6]. Although, collectively these studies implicate *LRRTM3* SNPs, haplotypes or multilocus genotypes (MLGs) in risk for LOAD, to date there have not been any systematic fine-mapping analysis of the *LRRTM3* region in LOAD.

In this study, we first aimed to characterize the intracellular, biochemical and gene expression interactions between LRRTM3, APP and BACE1, given the potential functional role of LRRTM3 in promoting BACE1 processing of APP [4]. Second, we aimed to accomplish fine mapping of the *LRRTM3* locus by analyzing 69 SNPs in this region in a LOAD case-control cohort (1,567 LOADs and 2,082 controls) and by follow-up of the significant results in a second independent series (1,258 LOADs and 718 controls). Our findings demonstrate intracellular, biochemical and gene expression interactions between LRRTM3 and APP and BACE1 for the first time and also identify a region in the 5′UTR-Intron 1 of *LRRTM3* that has variants which associate with LOAD risk. The gene expression studies from the *in-vitro LRRTM3* knock-down model and human brains, reveal complex correlations between mRNA levels of *LRRTM3*, *CTNNA3*, *APP* and *BACE1.* These gene and protein interactions may ultimately influence APP metabolism and AD risk. These results have implications for future genetic and functional studies of this gene and its role in AD.

## Materials and Methods

### Ethics Statement

All studies involving human samples were approved by the Mayo Clinic Institutional Review Board and appropriate written, informed consent was obtained from all participants. All mouse studies conducted in this study were approved by the Mayo Clinic Institutional Animal Care and Use Committee.

### LRRTM3 siRNA

Anti-human *LRRTM3* siRNA (si-LRRTM3) and control siRNA (si-control; MISSION®siRNA) were purchased from Sigma-Aldrich. Initially, three different *LRRTM3* siRNAs were tested (SASI_Hs02_00369484 = siRNA_1, SASI_Hs01_00163674 = siRNA_2, SASI_Hs01_00163676 = siRNA_3) and given its superior knock-down effect, SASI_Hs01_00163676 was chosen for all experiments in this study (**Text S1, Figures S1–S2**).

SH-SY5Y human neuroblastoma cells were stably transfected with human wild-type APP construct (SH-SY5Y-APP695wt). The same batch of SH-SY5Y cells were split and transfected with 50 pmols of either si-LRRTM3 or si-control with four separate transfections per treatment group, using TransFectin lipid reagent (BioRad) according to manufacturer’s protocols. After 24 hours, cells and media were harvested for Western blot analysis, Aβ40, Aβ42, sAPPα and sAPPβ ELISA experiments.

### ELISAs

Following harvesting, media from SH-SY5Y cells were submitted to ELISA measurements for Aβ40, Aβ42, sAPPα and sAPPβ. Aβ40 and Aβ42 levels in the media were measured using a sandwich ELISA system. Aβ40 ELISA system used 33.1.1 antibody (epitope: Aβ1–16) for capture and horse-radish peroxidase-conjugated 13.1.1 (13.1.1-HRP; epitope: Aβ35–40) as the detection antibody. For Aβ42 measurements, 2.1.3 antibody (epitope: Aβ35–42) was used for capture and 4G8-HRP (Covance®, epitope: Aβ17–24) for detection. Apart from 4G8, all other antibodies were originally manufactured by Mayo Clinic. Media was diluted 1∶5 in EC buffer for the Aβ40 measurements, but not for Aβ42. The Aβ ELISAs were done in duplicate and the average values were used in the statistical analyses. Optical density (OD) values for all measurements were obtained using the Softmax program. Absolute concentrations for the samples in femtomoles per milliliter unit were determined by comparison of their ODs to those of known concentrations of synthetic Aβ40 and Aβ42 peptides.

For endogenous mice brain Aβ measurements, the Mayo ELISA system utilizing antibodies 13.1.1 (capture) and 32.4.1-HRP (detection Aβ 1–16) were used for Aβ40 and the Wako ELISA system was used for Aβ42.

For sAPPβmeasurements, the BetaMark™ chemiluminescent kit from Covance® was utilized according to manufacturer’s protocols. All samples were diluted 1∶7 in EC buffer, then measured, using single measurement per experiment. sAPPα was measured using a commercial ELISA from IBL® per manufacturer’s instructions and as duplicates, using 1∶15 dilution in EC buffer.

For all ELISAs, si-LRRTM3 and si-control treated groups were assayed in a single batch and on the same ELISA plate. Each experiment was repeated at least one additional time.

Non-parametric Mann-Whitney test was used to assess significance of difference in results between si-LRRTM3 and si-control treated samples, using StatsDirect 2.7.8.

### Western Blot Analysis

Protein was extracted from harvested cells using Lysis Buffer (150mM NaCl, 50mM Tris pH 7.5, 0.1% Triton). After detection of the total protein concentration using Thermo BCA protein assay, equal amounts of protein for each treatment group were separated on a NuPage® 4–12% Bis-Tris gel (Invitrogen) and electrotransferred to Immobilon P membrane (Millipore, Bedford, MA) at 30 V for 1.5 hr. Membranes were blocked in 5% milk TBST and labeled overnight at 4°C with primary antibody CT20 against C-terminal domain of APP (gift from Pritam Das, Mayo Clinic Florida). Blots were incubated with HRP-linked secondary antibody for 1 hour (Invitrogen), and protein bands were detected using Western Lightning®Plus-ECL (Perkin Elmer). Equivalent sample loading was confirmed by probing the blots with the anti-GAPDH antibody (Pierce/Thermo Scientific).

### Immunocytochemistry

The intracellular localization of LRRTM3, APP and BACE1 was assessed in our SH-SY5Y-APP695wt monoclonal cell line, which was transfected with pCMV vector expressing a full length human LRRTM3 construct with a V5 tag, with or without co-transfection with a human full length BACE1 construct with an influenza hemagglutinin (HA) tag, also expressed within a pcDNA3 vector. Cells were seeded onto poly-D lysine coated cover slips, grown in Opti-MEM with 10% fetal bovine serum and transfected with Lipofectamine 2000 (Invitrogen) 24 hours after plating. Four hours post-transfection, the media was removed and replaced with fresh media containing 2 µl/ml baculovirus BacMam 2.0 (Invitrogen) expressing a GFP-tagged protein specific for one of the following organelles (targeted protein, catalog number): Early Endosomes-GFP (Rab5a, C10586, Invitrogen CellLight®Reagents), Golgi-GFP (N-acetylgalactosaminyltransferase 2, C10592) or Lysosomes-GFP (lysosomal associated membrane protein 1, O36228). After 24 hours, the cells were fixed in 4% paraformaldehyde, permeabilized, and blocked for 1 hr in 5% milk/PBS. Following incubation of primary antibody for 1 hour, cells were washed and incubated in conjugated secondary antibody for 1 hour. The following primary/secondary antibodies were used: LRRTM3: anti-V5/red Alexa Fluor 594 goat anti-mouse (double stains) or red Alexa Fluor 568 donkey anti-mouse (triple stains); BACE1: anti-HA/magenta Alexa Fluor 647 goat anti-rabbit; APP: CT-20/green Alexa Fluor 488 goat anti-rabbit (double stains) or magenta Alexa Fluor 647 goat anti-rabbit (triple stains); organelles: GFP fluorescence. All antibodies were from Invitrogen, except CT20. All antibodies were diluted 1∶1000 in 5% milk/PBS, except, CT20 which was diluted 1∶250.

We also performed confirmatory experiments for early endosomal localization of LRRTM3, by transfecting the SH-SY5Y-APP695wt cells with pcDNA6.2 vector expressing a full length human LRRTM3 construct with a GFP tag, and using an alternative endosomal stain with EEA1 (early endosome antigen 1) antibody 1∶1000 (BD Transduction 610456) and Alexa Fluor 568 donkey anti-mouse 1∶1000 (Invitrogen A10037; red) secondary antibody.

Nuclei were stained with DAPI (Molecular Probes). Immunostained cells were mounted using Fluoromount-G (SouthernBiotech) and imaged using the Zeiss LSM510 Laser Scanning Meta confocal microscope. The distribution of APP and LRRTM3 in neurons were detected by assessment of primary cortical neuronal cultures that were prepared from Tg2576 mice that expresses the Swedish mutant of APP (APPK670N,M671L) at high levels in the brain [18]. Primary cortical neuronal cultures were prepared from P2 mice and plated onto poly-D lysine coated cover slips, which were maintained in neurobasal media containing 2% B27 supplement, 2mM glutamine and gentamicin. To ensure robust transgene expression, we used an adeno-associated virus (rAAV1) mediated neuronal transduction method [19], [20]. We generated a viral construct expressing a human full-length LRRTM3 cDNA (NM_178011) with a V5 tag at the amino terminus. Neuronal cells were transduced at day 7 at a viral titer of 7.5×10^11^ gc/mL. After an additional 2 days, cells were fixed in 4% paraformaldehyde, washed and then permeabilized with 0.2% Triton X-100.

Neuronal cultures were incubated for 1 hour with primary antibodies V5 (Sigma; 1∶1000) to detect LRRTM3 and CT20 (1∶250) to detect APP, washed and then incubated with green Alexa Fluor 488 goat anti-mouse and red Alexa Fluor 594 goat anti-rabbit secondary antibodies to visualize LRRTM3 and APP, respectively. Images were acquired with the Zeiss LSM510 Laser Scanning Meta confocal microscope.

### Co-immunoprecipitation (co-IP) Experiments

Cultured human embryonic kidney HEK293T cells were grown for 24 hours (hrs) on 10 cm plates to 70% confluency and then transfected using Lipofectamine 2000 (Invitrogen) with the indicated expression constructs. Transfection media was removed after 4 hrs and replaced with fresh Opti-Mem with 10% FBS. After an additional 24 hrs the cells were harvested and lysed in IP buffer (150mM NaCl, 50mM Tris pH 7.5, 0.1% Triton) and total protein concentration was determined for each lysate. Immunoprecipitation (IP) protocol was then followed according to the manufacturer’s instructions (Immunoprecipitaion Kit- Dynabeads®Protein G (100.07D Invitrogen)). Briefly 1ug of either V5 Invitrogen P/N46-1157 (LRRTM3), GFP abcam ab6556 (BACE1) or CT20 (APP) antibody was bound to Dynabeads Protein G and incubated for 30 minutes with the protein lysate before being extensively washed. Additional IgG, IgM controls were included to ensure lack of non-specific binding of protein complexes. Eluted immunocomplexes were resolved on a 4–12% NuPage Bis-Tris gel (Invitrogen) and Western blot assays performed with the indicated primary antibody (1∶3000 dilution) and appropriate HRP conjugated secondary antibody (1∶3000 dilution/Invitrogen anti-rabbit NIF824,anti- mouse NIF825).

### Gene Expression Studies

#### *In-vitro* model

H4 human neuroglioma cells overexpressing wild type APP were transfected with three different anti-LRRTM3 siRNAs discussed above or si-control. Twenty-four hours post-transfection, total RNA extracted from harvested cells and was submitted to quantitative PCR (qPCR) to measure levels of LRRTM3 (Hs01060657_m1), BACE1 (Hs01123242_m1), CTNNA3 (Hs00203156_m1) and APP (Hs01552283_m1). Control genes were also measured (Hs99999905_m1 = GAPDH; Hs02800695_m1 = HPRT). TaqMan chemistry was utilized for the qPCR assays. Each experiment was replicated 2–6 times and measured in quadruplicate. All expression levels were normalized to HPRT. Expression levels relative to one of the replicates for the si-control treatment were calculated. The difference in these relative expression levels for each gene between the treatment groups was evaluated by Kruskall-Wallis test in StatsDirect (v2.7.8).

#### Human brains

Human brain expression levels for *LRRTM3*, *BACE1*, *CTNNA3*, *APP* and control genes were obtained from an autopsied cohort which was assessed in a recently published brain expression GWAS (eGWAS), where the methodology is described in detail (eGWAS) [21], [22]. Briefly, expression levels of 24,526 transcripts were measured from the cerebellum and temporal cortex of autopsied brains from subjects with pathologic AD (cerebellar n = 197, temporal cortex n = 202) and those with other brain pathologies (non-AD, cerebellar n = 177, temporal cortex n = 197). Total RNA extraction and QC were done using the Ambion RNAqueous kit and Agilent 2100 Bioanalyzer, respectively, according to published methods. Whole genome DASL expression microarrays (Illumina, San Diego, CA) were used for the transcriptome measurements of RNA samples that were randomized across chips and plates using a stratified approach to ensure balance with respect to diagnosis, age, sex, RNA Integrity Numbers (RIN) and *APOE* genotype. Raw probe-level expression data exported from Genome Studio software (Illumina Inc.) were preprocessed with background correction, variance stabilizing transformation, quantile normalization and probe filtering using the lumi package of BioConductor (Du et al., Bioinformatics, 2008; Lin et al., Nucleic Acid Res, 2008). Preprocessed probe transcript levels were used in the downstream analysis. The following probes were analyzed: *LRRTM3* (ILMN_2053334), *CTNNA3* (ILMN_2131732), *BACE1* (ILMN_2320349), *APP* (ILMN_2404063). All four probes had signals detectable above background for >75% of the samples from both brain regions and all diagnostic categories, with the exception of *CTNNA3*, which was detectable in 50–75% of the cerebellar samples.

Multivariable linear regression analyses were conducted, where expression levels for *LRRTM3* were used as the covariate, and those for the other gene were used as the dependent variable. Additional covariates that were utilized to correct for technical or biological variables include APOE ε4 dosage, age at death, sex, PCR plate, RIN, (RIN-RINmean)^2^, as well as expression levels of genes that are specific for the main five cell types present in the central nervous system (CNS), namely *ENO2* for neurons (ILMN_1765796), *GFAP* for astrocytes (ILMN_1697176), *CD68* for microglia (ILMN_2267914), *OLIG2* for oligodendrocytes (ILMN_1727567) and *CD34* for endothelial cells (ILMN_1732799). These 5 expression levels were included to account for neuronal loss, gliosis and/or vascular tissue in the assessed brain regions. All analyses were done for the cerebellar and temporal cortex gene expression levels, separately. All analyses were conducted in the combined AD and non-AD subjects, where AD diagnosis was included as a covariate. The statistical package StatsDirect was utilized for the multivariable linear regression analyses (v2.7.8).

#### LRRTM3 knock-out mice brains

We obtained *LRRTM3* knock-out mice from the the Mutant Mouse Regional Resource Center (MMRC). The mouse strain used for this research project, B6;129S5-Lrrtm3tm1Lex/Mmcd, identification number 032451-UCD was donated to the MMRRC by Genentech, Inc. Mouse hemibrains from *LRRTM3* knock-out, heterozygote and wild type animals were subjected to total RNA extraction using PureLink RNA Mini Kit Ambion, followed by qPCR to measure levels of endogenous mouse brain *LRRTM3* (Mm00618457_m1), *BACE1* (Mm00478664_m1), *CTNNA3* (Mm00617137_m1), *APP* (Mm01344172_m1), in addition to other *LRRTM*s (LRRTM1 = Mm00551337_g1, LRRTM2 = Mm00731288_s1) and control genes (GAPDH = Mm99999915_g1, HPRT = Mm0046968_m1). TaqMan chemistry was utilized for the gene expression assays. Control genes were used for normalization of gene expression levels. Three animals were used per each *LRRTM3* genotype, and each brain expression assay was done in quadruplicate. Mice brains were obtained at post-natal day 4 (P4) and also at ∼5.3 months of age. Endogenous mice Aβ ELISAs were done at P4 using mice hemibrains.

### Genetic Association Studies

#### Subjects and samples

Unrelated subjects from six independent LOAD case–control series, consisting of Caucasians with an age-at-diagnosis (LOAD), evaluation (elderly controls) or death (autopsy series) ≥60 years, were utilized in this study (3,166 LOAD vs. 3,261 controls; **Table S1 in File S1**). Three series were genotyped and analyzed for all of the SNPs as the exploratory cohort (Cohort 1). Cohort 1 series were collected at Mayo Clinic in Jacksonville, Florida (JS: 591 LOADs, 593 controls), Rochester, Minnesota, (RS: 553 LOADs, 1,374 controls) and an autopsy-confirmed series from the Brain Bank at Mayo Clinic Florida (AUT: 576 LOADs, 363 controls). The replication cohort (Cohort 2) consisted of subjects from the National Cell Repository for Alzheimer’s Disease [22] (NCRAD: 702 LOADs, 209 controls), Caucasian series from Poland [23] (PS: 483 LOADs, 189 controls), and from Norway [24] (NW: 261 LOADs, 533 controls). All clinical LOAD subjects had a diagnosis of probable or possible AD and all autopsied LOAD subjects of definite AD made according to NINCDS-ADRDA criteria [25]. All controls from the clinical Caucasian-American series had a clinical dementia rating score of 0. All autopsied LOAD brains had Braak scores of ≥4.0. Brains employed as controls had Braak scores of ≤2.5 but most had pathologies unrelated to AD that included vascular dementia, frontotemporal dementia, dementia with Lewy bodies, multi-system atrophy, amyotrophic lateral sclerosis, and progressive supranuclear palsy [26]. The autopsy cohort, which includes these non-AD control subjects with other pathologies have been utilized in other AD genetic association studies, including the Mayo LOAD risk GWAS [26], where it yielded results similar to the clinical LOAD case-control series. Nonetheless, it is theoretically possible that AD risk variants that also influence some of the other non-AD pathologies may not be picked up in this cohort, potentially leading to false-negatives. All DNA samples were isolated from peripheral blood, with the exception of samples in the autopsy series where DNA was isolated from donated brain tissue, as described in previous publications [22], [23], [24]. This study was approved by the appropriate institutional review board and appropriate informed consent was obtained from all participants.

#### SNP genotyping

Sixty-nine SNPs encompassing the region from the 5′ untranslated region (5′ UTR) of *LRRTM3* to its 3′ UTR were genotyped using either the Sequenom platform (Sequenom Inc., San Diego, CA, USA) [27] or TaqMan® SNP Genotyping Assay (Applied Biosystems, Foster City, CA). All variants had Hardy-Weinberg disequilibrium p values >0.0001 in controls, minor allele frequencies of >0.01 and genotyping rates >90% in each of the tested cohorts. All 69 SNPs were genotyped in the exploratory Cohort 1. The eight SNPs that constitute haplotype block 1, which form multilocus genotypes (MLGs) with suggestive global LOAD risk association in this cohort, were also genotyped in Cohort 2.

#### Statistical analysis

Single SNP analyses were done within PLINK [28] using logistic regression approach and an additive model, controlling for age at diagnosis/evaluation/death, sex, APOE4 dosage (0, 1, 2) and series. Each cohort was assessed individually and then jointly. Haplotype blocks were determined using the solid spine of linkage disequilibrium (LD) approach within Haploview program [29] and the SNP genotypes for all 69 SNPs from Cohort 1 series. The SNP haplotypes for this cohort were submitted to association analyses with LOAD risk using an additive model, and controlling for the covariates above, within Haplo.Stats [30]. Multilocus genotype analyses (MLGs) were done as previously described [31]. MLGs are defined by the combination of minor allele counts at all of the SNP loci, where each locus has 0, 1 or 2 minor alleles. Use of MLGs allows assessment of the genotypes at multiple SNP loci in a combined fashion and does not depend on individual haplotype estimations. After determination of the MLGs for SNPs within each LD block, for each subject, association with LOAD risk was tested. The MLGs within each LD block were tested by inclusion in a logistic regression model, where each subject gets a “1″ for the MLG that they have and “0″, otherwise. The most common MLG is used as the reference group. Age, sex, APOE4 dosage and series were again included as covariates. MLGs for SNPs within all LD blocks, except 1, were genotyped in Cohort 1 only. Those in LD block 1 were tested separately for Cohorts 1 and 2, as well as jointly. An additional MLG association analysis for block 1 SNPs was also conducted for all USA-Caucasian series (i.e. JS, RS, AUT, NCRAD). The p values and odds ratios (ORs) of association for each MLG as well as global significance for the model including all MLGs were determined within the StatDirect statistical package. Global significance was detected by comparison of the full model, including all covariates and MLGs to the model that only includes the covariates, where the difference between the chi squared test statistic based on the deviance likelihood ratios and degrees of freedom between the two models, were used to obtain the global p values for the MLGs.

## Results

### LRRTM3 Knock-down by siRNA Decreases BACE1 Processing Products of APP

Human neuroblastoma SH-SY5Y cells with stable overexpression of wild type APP (SH-SY5Y-APP695wt) were transfected with either anti-LRRTM3 siRNA (si-LRRTM3, SASI_Hs01_00163676, Sigma) or control (si-control, SIC001 MISSION Universal Negative Control#1, Sigma). The harvested media was assessed by ELISA measurements of Aβ40, Aβ42, sAPPα and sAPPβ and protein harvested from cells was analyzed for APP by Western blot analysis. Aβ40 levels were significantly lower in the si-LRRTM3 (mean±SEM = 441.0 fmol/ml ±8.5) vs. si-control (528.1 fmol/ml ±9.1) treated group (p = 0.03). Similarly, Aβ42 levels were also significantly lower in the si-LRRTM3 (173.0 fmol/ml ±7.4) vs. si-control (235.0 fmol/ml ±6.7) groups (p = 0.03). The secreted N-terminal BACE1 cleavage product of APP, sAPPβ, was also decreased significantly in the si-LRRTM3 (6667.7 pg/ml ±246.7) vs. si-control (8069.7 pg/ml ±576.1) group (p = 0.03). There was, however no difference in the α-secretase N-terminal cleavage product of APP, sAPPα, in the si-LRRTM3 (553.1 ng/ml ±27.2) vs. si-control (577.6 ng/ml ±24.3) groups (p = 0.9). Likewise, APP levels did not show any difference on Western blot analysis of the two groups.

### LRRTM3, APP and BACE1 Co-localize in Early Endosomes

**Figure 1:**
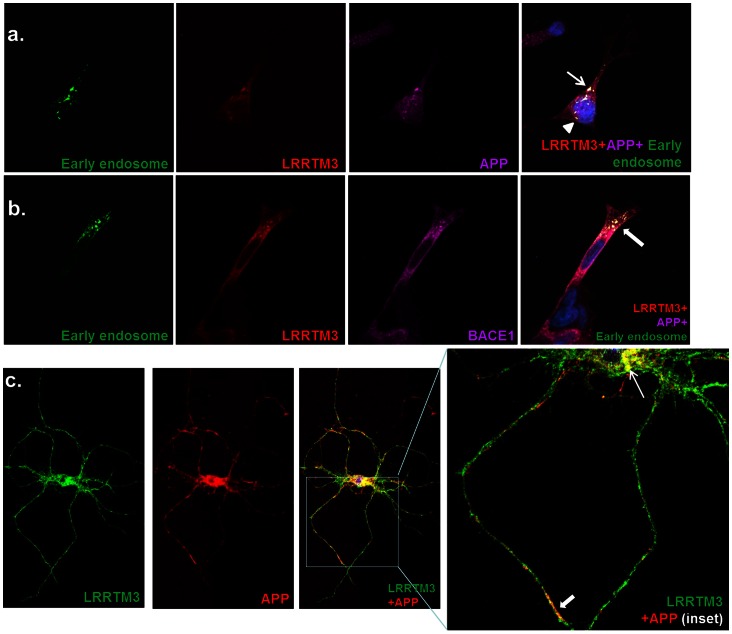
Co-localization of LRRTM3 and a) APP in early endosomes, b) BACE1 in early endosomes, c) APP in primary neurons. For a and b, SH-SY5Y-APP695wt cells were transduced with baculovirus expressing fused early-endosomal protein Rab5a and GFP. These cells were transfected with LRRTM3-V5 (a and b) and also with BACE1-HA (b). Results of staining with GFP fluorescence indicative of Rab5a expression (early endosomes, green); anti-V5 (LRRTM3, red); and either CT20 in a (APP, magenta) or anti-HA in b (BACE1, magenta) are shown with overlay of the three stains in the last panels of a and b. Co-localization of APP, LRRTM3 and early endosomes is visualized as white punctate intracellular structures in the last panel of a(arrow and arrowhead). Co-localization of APP, BACE1 and early endosomes is visualized as white punctate intracellular structures in the last panel of b. (Magnification: ×63). For c, primary neuronal cultures from Tg2576 transgenic mice transduced with rAAV-LRRTM3-V5 were stained with anti-V5 (LRRTM3, green) and CT20 (APP, red). Overlay of the two stains reveals co-localization of LRRTM3 and APP visualized as yellow puncta in the cell body (thin arrow) and neuronal process (thick arrow). (Magnification: ×100).

SH-SY5Y-APP695wt cells transiently transfected with LRRTM3 were transduced with baculovirus that expresses a fusion protein of an organelle marker and GFP. LRRTM3 co-localizes with the early endosomal marker (**Figure 1** **, Figure S3**), but not with lysosomes (**Figure S4**) or Golgi apparatus (**Figure S5**). When these cells were also stained for APP, co-localization of LRRTM3 and APP in punctate intracellular structures (**Figure 1a** **, Figure S6**), which also express the early endosomal marker is evident. APP also localizes within early endosomes (**Figure 1a** **, Figures S7–S8**), which is consistent with the literature [32].

Likewise, SH-SY5Y-APP695wt cells with transient transfection of both LRRTM3 and BACE1 and expressing the early endosomal marker, reveal localization of BACE1 to early endosomes (**Figure 1b**), which is known [32], as well as co-localization of LRRTM3 and BACE1 within early endosomes.

Finally, primary cortical neuronal cultures from Tg2576 mice expressing the Swedish mutant form of APP in the brain [18], were transduced with rAAV1 expressing human full-length LRRTM3 with V5 tag and stained for both LRRTM3 and APP (**Figure 1c**). The rationale for using Tg2576 mice is to enable assessment of neuronal co-localization of human APP with human LRRTM3. We determined that human LRRTM3 and APP co-localize in both the cell body and neuronal processes in these primary neuronal cultures.

### Co-immunoprecipitation (Co-IP) of LRRTM3, APP and BACE1

**Figure 2:**
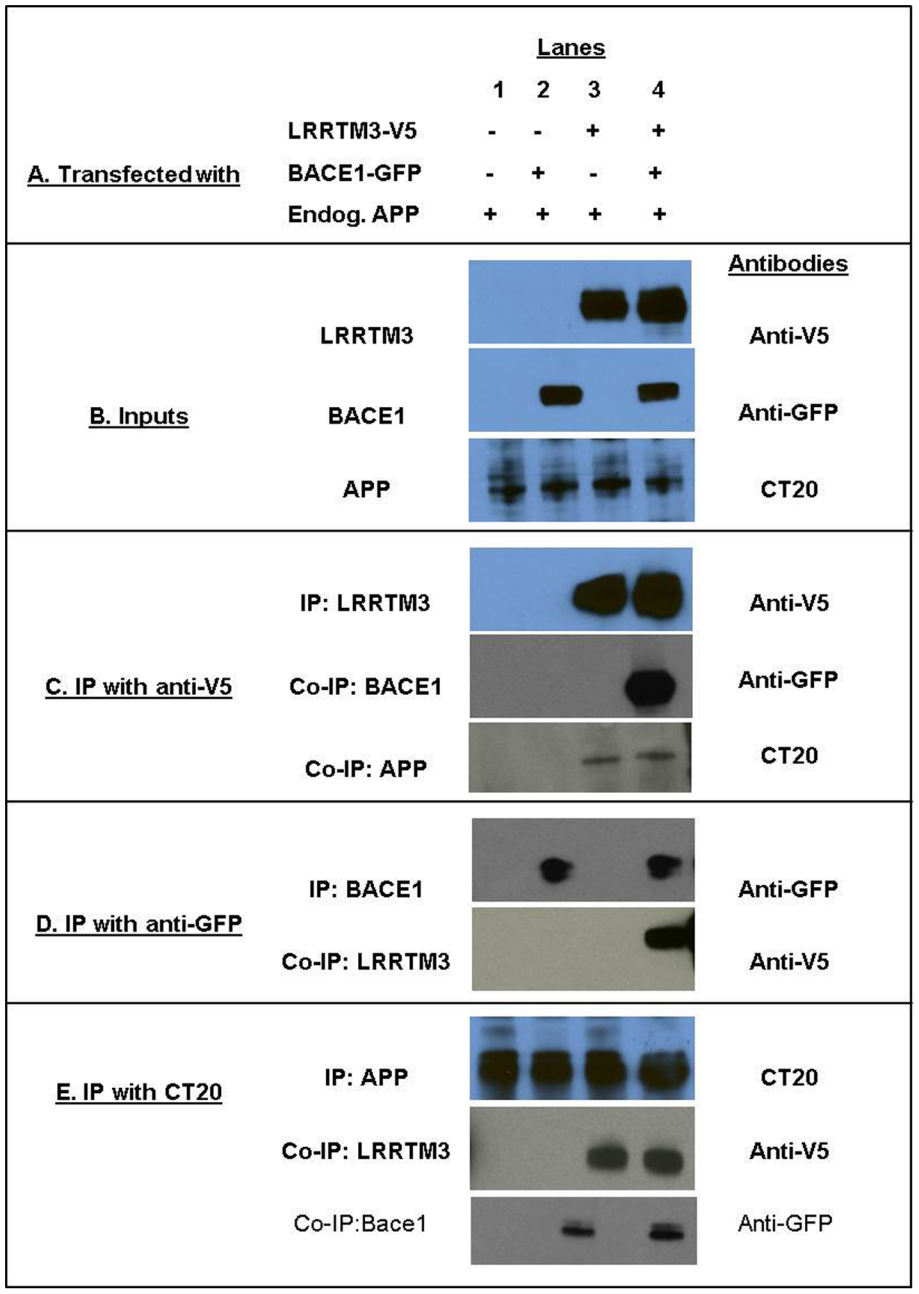
Co-IP of BACE1 and endogenous APP with LRRTM3 in HEK293T cells. LRRTM3-V5 and BACE1-GFP were transfected into HEK293T cells as shown in (a) and as in inputs (b). Protein lysates from these cells and negative controls without overexpression were immunoprecipitated using c. anti-V5 (LRRTM3), d. anti-GFP (BACE1) or c. CT20 (APP) antibodies. In the IP with anti-V5 (c), Western blot assays using anti-GFP or CT20, demonstrate co-IP of BACE1 and endogenous APP with LRRTM3. Similarly, LRRTM3 staining is clearly demonstrated in IPs for BACE1 (d) and for APP (e), indicating reverse co-IP of LRRTM3 with both proteins. Presence (+) or absence (−) of each of the three proteins for the depicted experiments are shown in (a) for each of the experimental conditions within the four lanes. Antibodies used in the Western blots are listed to the right of each figure. Proteins that are IP’ed or co-IP’ed are shown to the left of each figure.

To determine whether there is a biochemical interaction between LRRTM3, APP and BACE1 we co-transfected HEK293T cells with constructs expressing LRRTM3-V5 and BACE1-GFP (**Figure 2**). The transfection conditions (**Figure 2a**) and input amounts of each of the three proteins (**Figure 2b**) are shown. We performed IP with either anti-V5 (**Figure 2c**), anti-GFP (**Figure 2d**) or CT20 (anti-APP, **Figure 2e**), and as expected, detected strong LRRTM3 or BACE1 signals where these constructs were transfected and IP’ed, as well as endogenous APP expression for all conditions.

When the eluted immunocomplexes from the IP with LRRTM3 were probed for BACE1 or APP, it was evident that both proteins co-IP’ed with LRRTM3 (**Figure 2c**). Similarly, LRRTM3 could be detected from the immunocomplexes that were IP’ed for BACE1 (**Figure 2d**) or for endogenous APP (**Figure 2e**).

### Gene Expression Studies

**Figure 3:**
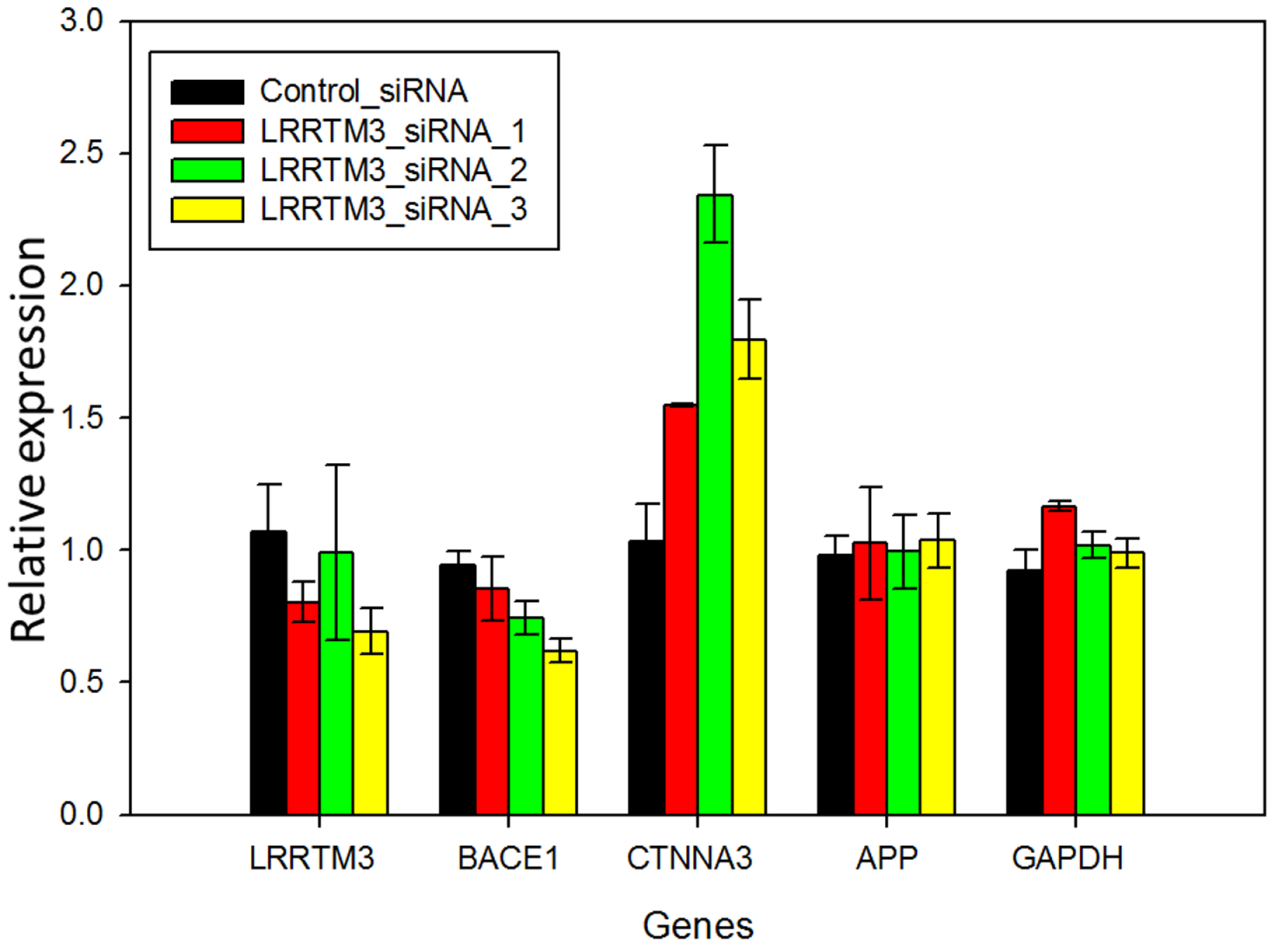
Relative expression levels of genes in H4 cells treated with three anti-*LRRTM3* and a control siRNA. Bar graphs depicting mean relative gene expression levels and error bars representing the standard deviations obtained from the averages of 2–6 experiments where each experiment is assessed in quadruplicate. Relative expression values are obtained by the delta delta Ct method, where HPRT is utilized as the control gene (delta Ct) and all results are normalized to one of the control wells (delta delta Ct). Relative expression values (2?(-delta delta Ct)) are plotted on the y-axis. The different siRNA treatment groups are color-coded as shown in the inset. The genes with expression level measurements are shown in groups, with gene names depicted on the x-axis.

To investigate whether the siRNA knock-down of *LRRTM3* influenced expression levels of *BACE1*, *APP* or its catenin counterpart *CTNNA3*, we tested the three *LRRTM3* siRNAs and the control siRNA in H4 cells that stably overexpress wild type APP and measured expression levels of these genes. *LRRTM3* siRNA_1 (SASI_Hs02_00369484) and siRNA_3 (SASI_Hs01_00163676) clearly led to knock-down of this gene. *LRRTM3* expression results for siRNA_2 (SASI_Hs01_00163674) treatment experiments had a larger standard deviation precluding this conclusion (**Figure 3** **, Table S2 in File S1**). Nonetheless, all three *LRRTM3* siRNAs had a knock-down effect observed in the Westerns (**Figure S1**), although siRNA_3 had the biggest effect (**Figure 3** **, Figures S1–S2, Table S2 in File S1**). Testing of differences between the different treatment groups by Kruskall-Wallis non-parametric test revealed significant differences for *LRRTM3* gene expression levels (p = 0.016). We observed knock-down of *BACE1* and increase in *CTNNA3* expression levels, the trends of which were consistent especially for the siRNA_1 and siRNA_3 knocked-down *LRRTM3* levels. Significant treatment-group effects were observed for *BACE1* (p = 0.006) and *CTNNA3* (p = 0.004), but not for APP gene expression (p = 0.9).

We next investigated correlations between brain expression of *LRRTM3* and those of the same three genes, *BACE1*, *APP* and *CTNNA3*, using expression levels from ∼400 human brains utilized in our eGWAS [22]. All analyses were done while correcting for technical and biological variables, as well as for expression levels of genes that are specific for the five cell types present in the CNS, as described in the Methods. Interestingly, *LRRTM3* and *CTNNA3* levels are negatively correlated in the temporal cortex (p<0.0001, β = −0.24) similar to the trend in H4 cells, but show positive correlations in the cerebellum (p = 0.006, β = 0.18) (**Table 1** **, Figure S9**). Like the H4 cells, *LRRTM3* and *BACE1* levels are positively correlated in the cerebellum (p = 0.0003, β = 0.11), but do not show significant association in the temporal cortex (p = 0.27, β = 0.02). Since BACE1 levels were found to be higher in the temporal cortex of AD brains compared to non-ADs [33], any potential associations between *LRRTM3* and *BACE1* levels may be obscured in the AD temporal cortex. Indeed, when we repeated our analyses only in the non-AD brains, *LRRTM3* showed positive correlations in the temporal cortex of this subset, as well (p = 0.02, β = 0.07, 95%CI = 0.01–0.13), but not in the ADs (p = 0.7). *LRRTM3* and *APP* brain levels showed strong positive correlations in both temporal cortex (p<0.0001, β = 0.15) and the cerebellum (p<0.0001, β = 0.24), with similarity in the effect sizes between these two independent brain regions. There were two other probes for APP (ILMN_1653283 and ILMN_2404065), both of which showed significant positive correlations in the cerebellum and temporal cortex, except ILMN_2404065, which was not significant (p = 0.21) but had a positive trend in the temporal cortex (β = 0.04).

**Table 1:** Human brain expression correlations between levels of *LRRTM3*, and *CTNNA3*, *BACE1* or *APP*.

Outcome Variable	Covariate	Temporal cortex				Cerebellum			
		P	Beta	95%CI		P	Beta	95%CI	
ILMN_2131732_CTNNA3	ILMN_2053334_LRRTM3	<0.0001	−0.24	−0.36	−0.13	0.006	0.18	0.05	0.30
ILMN_2320349_BACE1		0.27	0.02	−0.02	0.07	0.0003	0.11	0.05	0.17
ILMN_2404063_APP		<0.0001	0.15	0.11	0.20	<0.0001	0.24	0.16	0.32

Multivariable linear regression analyses were conducted while controlling for technical variables (plate, RIN), biological variables (diagnosis, age, sex, APOE4 dose) and variables accounting for cell loss, gliosis and vascularity by including as covariates expression levels of genes highly expressed in neurons (*ENO2*), astrocytes (*GFAP*), oligodendrocytes (*OLIG2*), microglia (*CD68*) and endothelial cell (*CD34*). The brain levels of *CTNNA3*, *BACE1* or *APP* were used as the outcome variable in a model, which included the above covariates and *LRRTM3* brain expression levels. Gene expression levels were detected in both the temporal cortex and cerebellum, for which results are shown separately. The significance (p), effect size (Beta) and 95% confidence interval of the effect size (95%CI) of *LRRTM3* expression for each of the tested genes are shown. Significant results are highlighted. Negative beta reflects an inverse relationship and positive correlation have a positive beta.

**Figure 4:**
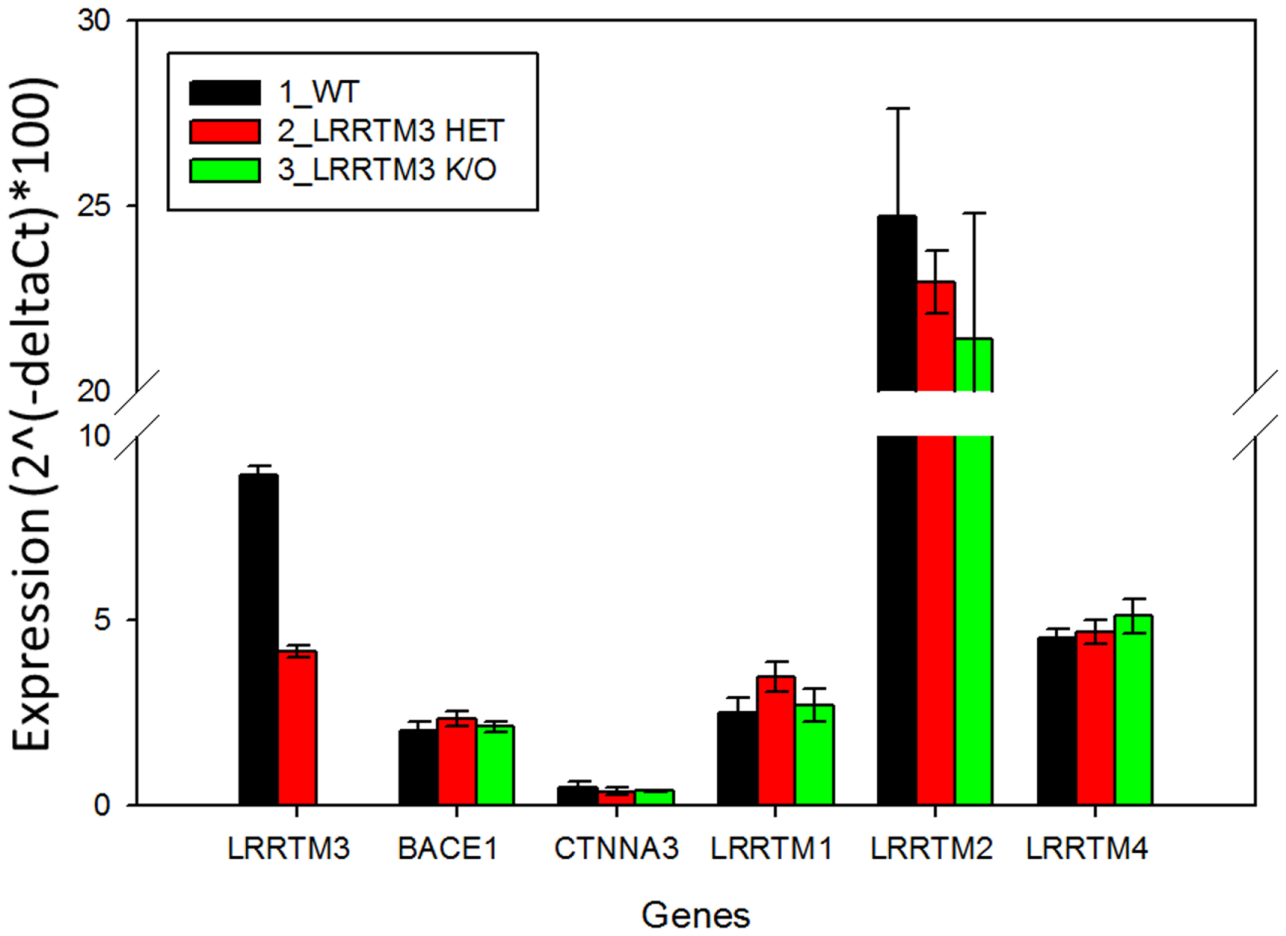
Expression levels of genes in brains of *Lrrtm3* knock-out, heterozygote and wild type mice. Bar graphs depicting mean gene expression levels and error bars representing the standard deviations obtained from the averages of 3 animals per genotypic group where expression levels from each mouse brain is assessed in quadruplicate. Expression values are obtained by the delta Ct method, where geometric mean of HPRT and GAPDH is utilized as the control gene expression values. Average expression values (2?(-delta Ct))x100 were plotted on the y-axis. The three mouse genotypic groups are color-coded as shown in the inset. The genes with expression level measurements are shown in groups, with gene names depicted on the x-axis.

Finally, we assessed endogenous mouse brain expression levels of *LRRTM3, BACE1, CTNNA3, APP* and the other LRRTMs (*LRRTM1, LRRTM2 and LRRTM4*) in *LRRTM3* knock-out (ko), heterozygote and wild type mice (**Figure 4**). We observed the expected ∼50% reduction of brain *LRRTM3* in the heterozygote and the absence of *LRRTM3* in the ko mice (p = 0.04), but there were no differences in the levels of any of the other tested genes at either P4 (**Figure 4** **, Table S3 in File S1**) or the later 5.3 month timepoints (data not shown). There were no differences in the P4 mouse brain endogenous Aβ40 or Aβ42 levels of the mice from the three *LRRTM3* genotypic groups (data not shown).

### Association of Genetic Variants at the LRRTM3 Locus with LOAD Risk

Single SNP analysis of 69 SNPs at the *LRRTM3* locus in the exploratory Cohort 1 composed of 1,720 ADs and 2,330 controls (**Table S1 in File S1**) revealed that none of the SNPs were significantly associated with LOAD risk after corrections for multiple testing (**Table S4 in File S1**). Haplotype analysis likewise did not reveal any significant associations (**Table S5 in File S1**). There were 7 haplotype blocks of SNPs in LD (**Figure S10**). Analysis of MLGs in Cohort 1 showed suggestive LOAD risk association for the multilocus SNP genotypes for LD block 1 variants (p = 0.06, **Table 2** **, Tables S5, S6 in File S1**) encompassing 5′UTR-Intron 1 regions of *LRRTM3*, but not for any of the MLGs in the other blocks. The SNPs in LD block 1 (**Figure S10**) were therefore genotyped and assessed in the replication Cohort 2 (1,446 ADs and 931 controls, **Table S1 in File S1**). Though they did not achieve significance in this replication cohort (p = 0.55), when Cohorts 1 and 2 were jointly assessed (3,166 LOAD and 3,261 controls), the global MLG association was significant (p = 0.036), with three MLGs achieving nominal significance (p<0.05) and five others that are suggestive (0.05<p<0.2). To investigate whether the presence of three series with different countries of origin in Cohort 2 could have led to decreased level of significance, secondary to potential increase in heterogeneity, we also investigated MLG association for LD block 1 variants in just the series from the USA (**Table 1** **,** **Table S4 in File S1**). Indeed, global MLG association was slightly better in the Caucasian-USA series (p = 0.02) despite smaller sample size (2,422 LOADs and 2,539 controls) than combined cohorts 1+2. Eight MLGs showed significant or suggestive LOAD risk association in the Caucasian-USA series, seven of which overlapped with the combined Cohort 1+2 analysis and five of which has improved significance. We note that while none of the individual MLGs would be significant after correcting for 31 tested MLGs, the global MLG associations do not require such a correction, so global p<0.05 is statistically significant.

**Table 2:** Multilocus genotype (MLG) analysis results.

	Cohorts 1+2 (N = 3166 ADs vs. 3261 controls)						Cohort 1 (N = 1720 ADs vs. 2330 controls)						Cohort 2 (1446 ADs vs. 931 controls)						USA Series Only (2422 ADs vs. 2539 controls)					
**MLG**	**CON (freq)**	**AD (freq)**	**P**	**OR**	**L95**	**U95**	**CON (freq)**	**AD (freq)**	**P**	**OR**	**L95**	**U95**	**CON (freq)**	**AD (freq)**	**P**	**OR**	**L95**	**U95**	**CON (freq)**	**AD (freq)**	**P**	**OR**	**L95**	**U95**
**MLG00002000**	**18 (0.006)**	**13 (0.005)**	**0.06**	**0.41**	**0.17**	**1.03**	**16 (0.008)**	**4 (0.003)**	**0.02**	**0.22**	**0.06**	**0.79**	2 (0.003)	9 (0.007)	0.7	1.5	0.2	10.4	**17 (0.007)**	**10 (0.004)**	**0.04**	**0.35**	**0.13**	**0.94**
MLG00011001	134 (0.047)	154 (0.053)	0.41	1.14	0.84	1.54	93 (0.044)	89 (0.056)	0.55	1.12	0.78	1.61	41 (0.055)	65 (0.05)	0.6	1.2	0.7	2.1	104 (0.045)	124 (0.055)	0.75	1.06	0.75	1.49
MLG00020002	354 (0.124)	349 (0.121)	0.80	1.03	0.83	1.28	279 (0.132)	195 (0.123)	0.84	0.97	0.75	1.26	75 (0.1)	154 (0.119)	0.4	1.2	0.8	1.8	304 (0.132)	281 (0.124)	0.94	0.99	0.78	1.26
MLG00101000	42 (0.015)	43 (0.015)	0.91	1.03	0.60	1.75	31 (0.015)	24 (0.015)	0.94	0.98	0.51	1.85	11 (0.015)	19 (0.015)	0.7	1.2	0.5	3.3	36 (0.016)	38 (0.017)	0.89	0.96	0.54	1.70
**MLG00110001**	228 (0.08)	215 (0.075)	0.39	0.89	0.69	1.15	149 (0.071)	114 (0.072)	0.85	0.97	0.71	1.33	**79 (0.106)**	**101 (0.078)**	**0.2**	**0.8**	**0.5**	**1.2**	163 (0.071)	164 (0.072)	0.99	1.00	0.74	1.34
**MLG00110002**	9 (0.003)	6 (0.002)	0.94	0.95	0.26	3.43	8 (0.004)	1 (0.001)	0.23	0.24	0.02	2.49	**1 (0.001)**	**5 (0.004)**	**0.2**	**5.4**	**0.5**	**58.0**	8 (0.003)	3 (0.001)	0.45	0.52	0.09	2.88
MLG00200000	40 (0.014)	37 (0.013)	0.65	0.88	0.50	1.54	26 (0.012)	18 (0.011)	0.86	1.07	0.52	2.19	14 (0.019)	19 (0.015)	0.3	0.7	0.3	1.6	32 (0.014)	28 (0.012)	0.69	0.88	0.46	1.68
MLG01010001	15 (0.005)	20 (0.007)	0.72	1.16	0.52	2.63	12 (0.006)	12 (0.008)	0.73	1.18	0.46	3.01	3 (0.004)	8 (0.006)	1.0	1.0	0.2	5.1	12 (0.005)	16 (0.007)	0.49	1.38	0.56	3.40
MLG01011011	9 (0.003)	4 (0.001)	0.42	0.56	0.14	2.28	7 (0.003)	3 (0.002)	0.74	0.77	0.17	3.61	2 (0.003)	1 (0.001)	0.3	0.2	0.0	4.3	8 (0.003)	4 (0.002)	0.54	0.64	0.15	2.72
MLG01020012	34 (0.012)	36 (0.013)	0.45	1.26	0.70	2.26	30 (0.014)	23 (0.015)	0.72	1.13	0.59	2.16	4 (0.005)	13 (0.01)	0.3	2.0	0.5	8.5	31 (0.013)	31 (0.014)	0.59	1.19	0.63	2.22
MLG01110011	8 (0.003)	9 (0.003)	0.57	1.42	0.42	4.76	8 (0.004)	6 (0.004)	0.72	1.25	0.36	4.40	0 (0)	3 (0.002)	1.0	NA			8 (0.003)	9 (0.004)	0.54	1.46	0.43	4.90
**MLG10020111**	**22 (0.008)**	**9 (0.003)**	**0.17**	**0.49**	**0.17**	**1.36**	21 (0.01)	4 (0.003)	0.10	0.36	0.11	1.21	1 (0.001)	5 (0.004)	0.5	2.9	0.1	73.5	**21 (0.009)**	**7 (0.003)**	**0.16**	**0.46**	**0.16**	**1.36**
**MLG11001110**	**137 (0.048)**	**158 (0.055)**	**0.08**	**1.31**	**0.97**	**1.77**	**102 (0.048)**	**94 (0.059)**	**0.06**	**1.40**	**0.99**	**1.99**	35 (0.047)	64 (0.049)	0.6	1.1	0.7	2.0	**112 (0.049)**	**136 (0.06)**	**0.05**	**1.39**	**1.00**	**1.93**
MLG11010002	11 (0.004)	17 (0.006)	0.65	1.24	0.49	3.16	6 (0.003)	7 (0.004)	0.54	1.49	0.42	5.35	5 (0.007)	10 (0.008)	1.0	1.0	0.3	4.0	7 (0.003)	7 (0.003)	0.86	1.12	0.33	3.82
**MLG11010012**	**12 (0.004)**	**8 (0.003)**	**0.05**	**0.36**	**0.13**	**1.00**	8 (0.004)	5 (0.003)	0.21	0.45	0.13	1.57	**4 (0.005)**	**3 (0.002)**	**0.1**	**0.3**	**0.0**	**1.4**	8 (0.003)	7 (0.003)	0.31	0.55	0.17	1.77
MLG11010111	716 (0.251)	731 (0.254)	REF				540 (0.256)	411 (0.259)	REF				176 (0.236)	320 (0.247)	REF				590 (0.256)	558 (0.246)	REF			
**MLG11011110**	24 (0.008)	16 (0.006)	0.20	0.61	0.29	1.30	15 (0.007)	10 (0.006)	0.58	0.76	0.29	1.98	**9 (0.012)**	**6 (0.005)**	**0.2**	**0.5**	**0.1**	**1.5**	16 (0.007)	12 (0.005)	0.58	0.78	0.32	1.91
**MLG11020111**	**74 (0.026)**	**96 (0.033)**	**0.10**	**1.38**	**0.94**	**2.01**	**48 (0.023)**	**53 (0.033)**	**0.09**	**1.50**	**0.94**	**2.39**	26 (0.035)	43 (0.033)	0.7	1.1	0.6	2.2	**51 (0.022)**	**72 (0.032)**	**0.03**	**1.63**	**1.05**	**2.53**
**MLG11020112**	8 (0.003)	9 (0.003)	0.18	2.07	0.71	6.01	**6 (0.003)**	**6 (0.004)**	**0.13**	**2.50**	**0.75**	**8.27**	2 (0.003)	3 (0.002)	1.0	1.1	0.1	9.7	**6 (0.003)**	**8 (0.004)**	**0.11**	**2.66**	**0.81**	**8.81**
MLG11100110	245 (0.086)	213 (0.074)	0.29	0.87	0.68	1.12	186 (0.088)	116 (0.073)	0.20	0.82	0.61	1.11	59 (0.079)	97 (0.075)	1.0	1.0	0.6	1.6	201 (0.087)	168 (0.074)	0.38	0.88	0.67	1.17
MLG11100111	7 (0.002)	5 (0.002)	0.77	0.82	0.22	3.03	4 (0.002)	4 (0.003)	0.67	1.39	0.30	6.46	3 (0.004)	1 (0.001)	0.3	0.2	0.0	3.4	NA					
MLG11110110	31 (0.011)	24 (0.008)	0.52	0.81	0.44	1.52	18 (0.009)	13 (0.008)	0.93	0.97	0.44	2.12	13 (0.017)	11 (0.008)	0.4	0.6	0.2	1.9	19 (0.008)	21 (0.009)	0.73	1.13	0.55	2.33
MLG12000210	7 (0.002)	11 (0.004)	0.40	1.59	0.53	4.72	4 (0.002)	6 (0.004)	0.36	1.93	0.47	7.99	3 (0.004)	5 (0.004)	0.8	1.2	0.2	6.3	5 (0.002)	8 (0.004)	0.53	1.53	0.40	5.82
MLG12010121	25 (0.009)	33 (0.011)	0.57	1.20	0.64	2.27	20 (0.009)	25 (0.016)	0.50	1.27	0.63	2.57	5 (0.007)	8 (0.006)	0.9	1.1	0.3	4.3	21 (0.009)	29 (0.013)	0.55	1.23	0.62	2.45
**MLG21010220**	**13 (0.005)**	**21 (0.007)**	**0.02**	**2.63**	**1.18**	**5.84**	**8 (0.004)**	**16 (0.01)**	**0.0035**	**4.03**	**1.58**	**10.29**	5 (0.007)	5 (0.004)	0.6	0.6	0.1	3.0	**9 (0.004)**	**21 (0.009)**	**0.003**	**3.93**	**1.62**	**9.53**
MLG22000111	12 (0.004)	16 (0.006)	0.93	1.04	0.43	2.50	7 (0.003)	6 (0.004)	0.71	1.26	0.37	4.24	5 (0.007)	10 (0.008)	0.7	0.8	0.2	2.7	7 (0.003)	8 (0.004)	0.49	1.51	0.47	4.89
**MLG22000121**	**7 (0.002)**	**18 (0.006)**	**0.05**	**2.71**	**0.98**	**7.47**	7 (0.003)	8 (0.005)	0.26	1.94	0.62	6.12	0 (0)	10 (0.008)	1.0	NA			**7 (0.003)**	**16 (0.007)**	**0.06**	**2.64**	**0.94**	**7.42**
MLG22000220	394 (0.138)	421 (0.146)	0.46	1.08	0.88	1.33	289 (0.137)	208 (0.131)	0.88	1.02	0.79	1.31	105 (0.141)	213 (0.164)	0.3	1.2	0.8	1.7	318 (0.138)	330 (0.146)	0.47	1.09	0.87	1.37
MLG22010220	99 (0.035)	87 (0.03)	0.67	0.92	0.64	1.32	76 (0.036)	48 (0.03)	0.54	0.87	0.56	1.35	23 (0.031)	39 (0.03)	0.9	1.0	0.5	2.0	83 (0.036)	68 (0.03)	0.64	0.91	0.61	1.36
MLG22010221	8 (0.003)	5 (0.002)	0.74	0.79	0.20	3.11	5 (0.002)	1 (0.001)	0.56	0.52	0.06	4.72	3 (0.004)	4 (0.003)	0.9	1.1	0.1	8.5	NA					
**MLG-rare**	**113 (0.04)**	**96 (0.033)**	**0.01**	**0.64**	**0.45**	**0.91**	**80 (0.038)**	**55 (0.035)**	**0.10**	**0.70**	**0.45**	**1.08**	**33 (0.044)**	**41 (0.032)**	**0.1**	**0.6**	**0.3**	**1.1**	**103 (0.045)**	**82 (0.036)**	**0.03**	**0.65**	**0.45**	**0.95**
**Global p**	**0.036**						**0.06**						0.55						**0.020**					

Logistic regression analysis with MLGs of 8 SNPs from Haplotype Block 1 are done controlling for age, sex, APOE4 dosage and series. Results from Cohorts 1, 2, the USA only cohorts and combined Cohorts 1+2 analyses are shown. CON = control and AD = Alzheimer's disease numbers and percentages for each MLG are shown. P = P values, OR = odds ratio, L95 = lower 95% and U95 = upper 95% confidence interval of OR for each MLG is shown for each analysis. REF = the most common MLG is used as the reference genotype. NA = results of MLGs where one group has 0 subjects or where there are less than 10 subjects in total are not available. MLGs with p<0.2 are bolded. Global p value of association for all MLGs are also shown. We note that while none of the individual MLGs would be significant after correcting for 31 tested MLGs, the global MLG associations do not require such a correction, so global p<0.05 is statistically significant. MLGs with total subject counts <10 in the combined Cohorts 1+2 are grouped into the MLG-rare group.

## Discussion

In this study we investigate functional and genetic role of LRRTM3 in AD risk. In our functional studies we demonstrate co-localization of LRRTM3, APP and BACE1 in early endosomes of human neuroblastoma cells; co-localization of LRRTM3 and APP in cell body and processes of primary neurons from Tg2576 mouse model [18] and co-IP of LRRTM3 with BACE1 and endogenous APP from HEK293T cells. We also confirm the prior results of decreased BACE1 cleavage products of APP in the setting of anti-LRRTM3 siRNA treatment of SH-SY5Y-APP695wt cells [4]. Unlike the original report, however, we find significant decrease of *BACE1* mRNA, upon treatment with siRNAs against *LRRTM3*. The discrepancy between our *LRRTM3* siRNA results in regards to lowering of *BACE1* levels and the Majercak et al. study [34], which did not observe any changes in *BACE1* levels upon lowering of *LRRTM3* could be due to several reasons. It is possible that technical differences could account for this: we utilized different siRNAs, evaluated expression changes in different cell types, utilizing different gene expression measurement approaches. It could be argued that the influence on *BACE1* of the *LRRTM3* siRNAs we utilized is an artifactual, off-target effect. We think this is unlikely, since we observe this effect with at least two of three siRNAs. Further, the positive correlations we detected between human brain levels of *LRRTM3* and *BACE1* raise the possibility that the levels of these genes might be co-regulated, although the correlations alone would not be sufficient for a definitive conclusion. Our findings suggest that the Aβ-lowering effect of *LRRTM3* knock-down in our *in-vitro* model, might be secondary to concurrent knock-down of *BACE1*, whereas Majercak et al. concluded that decreased *LRRTM3* levels might influence vesicle trafficking or signaling. In reality, more than one mechanism might be at play.

Indeed, we also observe significant increases in the α-catenin counterpart of *LRRTM3*, namely *CTNNA3*, upon knocking down of *LRRTM3 in-vitro*, which was not previously reported. The strong negative correlations of human brain *CTNNA3* and *LRRTM3* levels in the temporal cortex, is also consistent with the *in-vitro* knock-down results. Intriguingly, these negative correlations are only observed in the temporal cortex, which is the primarily affected region in AD, but in the cerebellum, which is an AD-unaffected region, strong positive correlations are observed between levels of these two genes. It is hypothesized that transcriptional regulation of nested genes may lead to either positive or negative correlations between gene levels, which might imply similar or diverse functions for the genes, respectively [35]. Investigation of the *LRRTM2/CTNNA1* and *LRRTM1/CTNNA2* pairs identified a common promoter with bi-directional transcription, which resulted in truncated CTNNA1 and CTNNA2 that are highly expressed in the CNS [36]. Collectively, these results underline the potential that changes in *LRRTM3* levels might also influence *CTNNA3* levels and that any downstream effects (such as those on Aβ metabolism) could be due to one or both of these genes. Although direct knock-down of *CTNNA3* did not influence Aβ levels in the Majercak et al. study, levels of *CTNNA3* were not evaluated in the *LRRTM3* knock-down model.

Our immunohistochemistry and biochemistry results suggest that APP, BACE1 and LRRTM3 co-localize to early endosomes and also have biochemical interactions. We acknowledge that these studies were conducted in paradigms where LRRTM3 was overexpressed due to lack of a robust antibody despite testing five different commercial antibodies and generating one ourselves (Supplementary Methods in **Text S1**). It is therefore theoretically possible that overexpression of LRRTM3 could have led to mislocalization. Despite this caveat, we have demonstrated not only localization of LRRTM3 in early endosomes, but also its lack of localization from lysosomes or Golgi apparatus. Furthermore, we have been able to detect both co-localization and biochemical interaction between endogenous APP with overexpressed LRRTM3. Additionally, though BACE1 was also overexpressed, we have been able to detect its appropriate localization to early endosomes and expected interactions with APP. While further studies are needed utilizing endogenous LRRTM3, our findings provide evidence for the first time, for interactions between LRRTM3, APP and BACE1. Interestingly, *LRRTM3* and *APP* levels show strong positive correlations in the human temporal cortex and cerebellum. Given that the *in-vitro* studies with siRNA against *LRRTM3* did not yield any changes in APP levels, the human brain transcriptome findings could imply an upstream regulatory factor for both genes. Alternatively, high levels of overexpressed *APP* in the *in-vitro* paradigm could mask any subtler changes in *APP* levels upon manipulating *LRRTM3* expression.

It should be noted that a recent study of *Lrrtm3* knock-out mice, by Laakso et al., did not identify any changes in either endogenous Aβ levels or processing of APP in mice crossed with the APP/PS1 mutant mice [37]. This finding is contradictory to the *in-vitro* findings in our study and others [6], [34]. One explanation is that all of the *in-vitro* findings are artifactual and due to off-target effects of siRNAs. While theoretically possible, given the observed effects on APP processing in multiple different paradigms with different siRNAs, alternative explanations also need to be considered. It is plausible that the influence on APP metabolism might be a species-specific function. Further, if the influence is secondary to regulatory changes in other genes, including *CTNNA3*, this regulatory machinery could differ between humans and mice. Indeed, unlike the findings in our *in-vitro* studies conducted in human-derived cells and human brain transcriptome, we did not see any gene expression changes in *App, Bace1* or *Ctnna3* in brains of *Lrrtm3* knock-out mice, although we used a different mouse strain than the Laakso et al. study [37]. Compensatory effects in knock-out animals is also a concern. While compensatory changes in other *Lrrtms* were not reported [37], it remains possible that there could be downstream modifications in other untested genes germaine to the APP metabolism. To further investigate the role of LRRTM3 in APP metabolism, additional studies focused on human cell lines and iPS cells are required. Further, thorough characterization of transcriptional changes in *Lrrtm3* knock-out mice, additional mouse models characterizing human *LRRTM3* and *CTNNA3* both separately and jointly are warranted.

Our genetic analysis revealed association of multilocus genotypes (MLG) in the 5′UTR-Intron 1 region of LRRTM3 with AD risk, marginally in the exploratory cohort, non-significant in the follow-up cohort, though significant overall and individual MLG associations in the combined cohort, supporting consistent trends in the second cohort. These associations were even more significant in the subset of Caucasian-American series, which might be due to decreased heterogeneity of this cohort. The lack of significant single SNP or haplotype associations, but significant MLG associations may be due to the presence of multiple functional genetic variants, interactions between alleles at the same SNP and between variants or a combination of these factors. Indeed, two other studies utilized the MLG approach with *LRRTM3* SNPs and showed association with AD risk. One study identified 5 *LRRTM3* SNPs, the MLGs of which formed three clusters, wherein variants of *PLAU*, *CDC2* and *ACE* showed significant AD risk association [15]. Another study identified two and three locus genotypes with SNPs in *LRRTM3*, *ACE* and *A2M* that associate with AD risk [17]. These findings suggest the presence of intreactions between variants at these genes, which have biological implications in APP metabolism. Although, our study only focused on MLGs within *LRRTM3*, our results can similarly be interpreted as being influenced by variant interactions at this region.

Our findings nominate the 5′UTR-Intron 1 region of *LRRTM3* (LD block 1) as having the most significant effect on AD risk. Two other studies identified AD risk variants in this region. Rs1925583, which showed significant AD risk association in APOE4 carriers [8], though not significant in our study, resides within the significant MLG block 1. Rs16923760, which associates with AD risk in a Caribbean-Hispanic series (OR = 0.74) [6], is in LD with rs4746648 (D’ = 1.0, r2 = 0.01) that has nominally significant AD risk association in our combined cohort (p = 0.02, OR = 0.86). Review of the individually significant MLGs does not lead to the identification of specific variants with risky or protective effects. This is likely to occur if there is heterogeneity, such that the SNPs at this MLG block may be tagging both risky and protective functional variants. Nevertheless, these results collectively suggest the presence of variants in the 5′UTR-Intron 1 region of LRRTM3 and should serve as a guide in the search of functional variation in this gene.

The *LRRTM3* variants in MLG block 1 are not in any LD with the top *CTNNA3* (*VR22*) variants that associate with Aβ levels [7] and AD risk in some studies. This suggests independent roles for these nested genes in AD. Any further possible functional and/or genetic interaction between *LRRTM3* and *CTNNA3* (*VR22*) remains to be elucidated.

In summary, our study identifies biochemical and immunocytochemical interactions between LRRTM3, BACE1 and APP; gene expression correlations between these genes and also CTNNA3; confirms the effect of LRRTM3 siRNA on reducing BACE1-cleavage products of APP and detects MLGs in the 5′UTR-Intron 1 region of LRRTM3 that associate with AD risk. Despite the strength of joint functional and genetic assessment of this gene, and detailed fine-mapping of this region in two cohorts, our study has some weaknesses. The co-localization experiments were conducted in paradigms where all three proteins and the co-IP experiments were done in models where LRRTM3 and BACE1 are overexpressed. This is a potential concern with all overexpression experiments, but may not be possible to avoid, often due to lack of appropriate antibodies, as was the case in our study. Nonetheless, the co-IP of endogenous APP with LRRTM3 and vice versa; the biologically consistent immunocytochemistry, biochemistry and siRNA data; alongside the genetic results provide collective support for a role of LRRTM3 in APP metabolism. Another weakness is the lack of significant MLG association in the second cohort, although improved significance in the combined cohorts 1+2 suggests consistent effects between the first and second cohorts. While it is not possible to discern the individual protective or risky variants from the MLG analysis, our results pinpoint to the specific 5′UTR-Intron 1 region of LRRTM3 for further studies.

LRRTM3, an evolutionarily conserved member of a synaptic protein family was also detected as a risk gene in autism [38], raising the possibility of a potential role of this gene in the neurodevelopment-neurodegeneration axis. There is significant precedence to pursue additional functional and genetic investigations of LRRTM3. Our results advocate downstream mechanistic studies that focus on interactions of LRRTM3 with APP, BACE1 and CTNNA3; and genetic studies that investigate the 5′UTR-Intron 1 of LRRTM3 for functional variants.

## Supporting Information

Figure S1 **Knock-down of LRRTM3 with different siRNAs.** SH-SY5Y cells with stable overexpression of LRRTM3 with a DDK tag (LRRTM3-DDK) were treated with three different siRNAs against LRRTM3 (lanes 1–3), control siRNA (lane 4) or not treated. Top and bottom panels show Western blot assays for LRRTM3 (anti-DDK) and GAPDH, respectively. The knock-down of LRRTM3 is evident with all three anti-LRRTM3 siRNAs, but not with the control siRNA. siRNAs in lanes 1–3 are as follows in order: SASI_Hs02_00369484, SASI_Hs01_00163674, SASI_Hs01_00163676.
(TIF)

Figure S2 **Dose-dependent knock-down of LRRTM3.** The fold change of *LRRTM3* levels in HEK293T cells are expressed with respect to lipofectamine control. The average normalized Ct values for each experimental group are utilized in the analyses. There were two experiments per group and 4 replicates per experiment. The ∼40% knock-down in *LRRTM3* expression was detected for *LRRTM3* siRNA SASI_Hs01_00163676 amount of 40 pmols or greater.
(TIF)

Figure S3 **Localization of LRRTM3 and early endosomes (example 2).** SH-SY5Y-APP695wt cells were transfected with LRRTM3-V5 and transduced with baculovirus expressing fused early-endosomal protein Rab5a and GFP. Results of staining with a. DAPI (nucleus); b. anti-V5 (LRRTM3); and c. GFP fluorescence indicative of Rab5a expression (early endosomes); d. overlay of a+b+c. There is abundant localization of LRRTM3 within the early endosomes. Magnification:×100.
(TIF)

Figure S4 **Localization of LRRTM3 and lysosomes.** SH-SY5Y-APP695wt cells transfected with LRRTM3-V5 and transduced with baculovirus expressing fused lysosomal protein Lamp1 and GFP. Results of staining with a. DAPI (nucleus); b. anti-V5 (LRRTM3); and c. GFP fluorescence indicative of Lamp1 expression (lysosomes); d. overlay of a+b+c. There does not appear to be localization of LRRTM3 within lysosomes. Magnification: ×100.
(TIF)

Figure S5 L**ocalization of LRRTM3 and Golgi apparatus.** SH-SY5Y-APP695wt cells transfected with LRRTM3-V5 and transduced with baculovirus expressing fused Golgi apparatus protein N-acetylgalactosaminyltransferase 2 and GFP. Results of staining with a. DAPI (nucleus); b. anti-V5 (LRRTM3); and c. GFP fluorescence indicative of N-acetylgalactosaminyltransferase 2 expression (Golgi); d. overlay of a+b+c. There does not appear to be localization of LRRTM3 within the Golgi apparatus. Magnification: ×100.
(TIF)

Figure S6 **Intracellular co-localization of LRRTM3 and APP (example 2).** SH-SY5Y-APP695wt cells were transfected with LRRTM3. Results of staining with a. DAPI (nucleus); b. CT20 (APP); and c. anti-V5 (LRRTM3); d. overlay of a+b+c. There is abundant co-localization of LRRTM3 with APP. Magnification: ×100.
(TIF)

Figure S7 **Co-localization of LRRTM3 and APP in early endosomes (example 2).** SH-SY5Y-APP695wt cells were transfected with LRRTM3-V5 and transduced with baculovirus expressing fused early-endosomal protein Rab5a and GFP. Results of staining with a. DAPI (nucleus); b. GFP fluorescence indicative of Rab5a expression (early endosomes); c. anti-V5 (LRRTM3); d.CT20 (APP); e. overlay of a+b+c; f. overlay of a+b+d; g. overlay of a+c+d; h.overlay of a+b+c+d. Co-localization of APP, LRRTM3 and early endosomes is visualized as white punctate intracellular structures in h and can also be seen in e-g. The two cells stained for APP and not with LRRTM3 or early endosomes clearly depict a different staining pattern than the cell in the middle of the field, which stains with all three proteins. Magnification: ×63.
(TIF)

Figure S8 **Co-localization of LRRTM3 and APP in early endosomes (example 3).** Same conditions and staining are used as Figure S5.
(TIF)

Figure S9 **Simple linear regression plots for** ***LRRTM3*** **vs. other gene brain expression level associations that are significant in** **Table 1** **.** Results for cerebellar expression level plots are shown first followed by those for the temporal cortex levels. The p values and correlation coefficients correspond to the plots depicted above them.
(DOCX)

Figure S10 **Linkage disequilibrium (LD) plot of 69 SNPs at the LRRTM3 locus.** Haploview 4.1 was utilized to obtain the LD plot depicting D’ values for all SNP pairs, using genotypes from Cohort 1. LD blocks are defined according to the solid spine of LD algorithm. SNP names are depicted at the top of the figure. Though, initially 71 SNPs were assessed, two SNPs which violated Hardy-Weinberg equilibrium in controls from either cohort were excluded from the analysis.
(TIF)

File S1 **Includes Tables S1–S6.**
(XLSX)

Text S1 **Supplementary text.**
(DOCX)
