# Supplementary figures and images for: LRRTM3 Interacts with APP and BACE1 and Has Variants Associating with Late-Onset Alzheimer’s Disease (LOAD)
Source: PLoS One. 2013 Jun 4;8(6):e64164. doi: 10.1371/journal.pone.0064164 (PMC3672107; doi:10.1371/journal.pone.0064164)

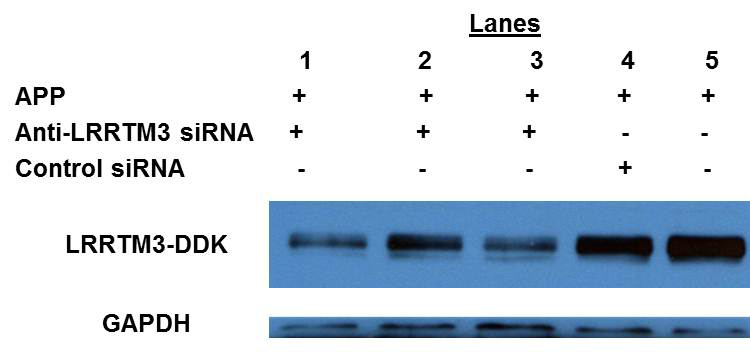

Supplement: Figure S1 — Knock-down of LRRTM3 with different siRNAs. SH-SY5Y cells with stable overexpression of LRRTM3 with a DDK tag (LRRTM3-DDK) were treated with three different siRNAs against LRRTM3 (lanes 1–3), control siRNA (lane 4) or not treated. Top and bottom panels show Western blot assays for LRRTM3 (anti-DDK) and GAPDH, respectively. The knock-down of LRRTM3 is evident with all three anti-LRRTM3 siRNAs, but not with the control siRNA. siRNAs in lanes 1–3 are as follows in order: SASI_Hs02_00369484, SASI_Hs01_00163674, SASI_Hs01_00163676. (TIF) [file pone.0064164.s001.tif]

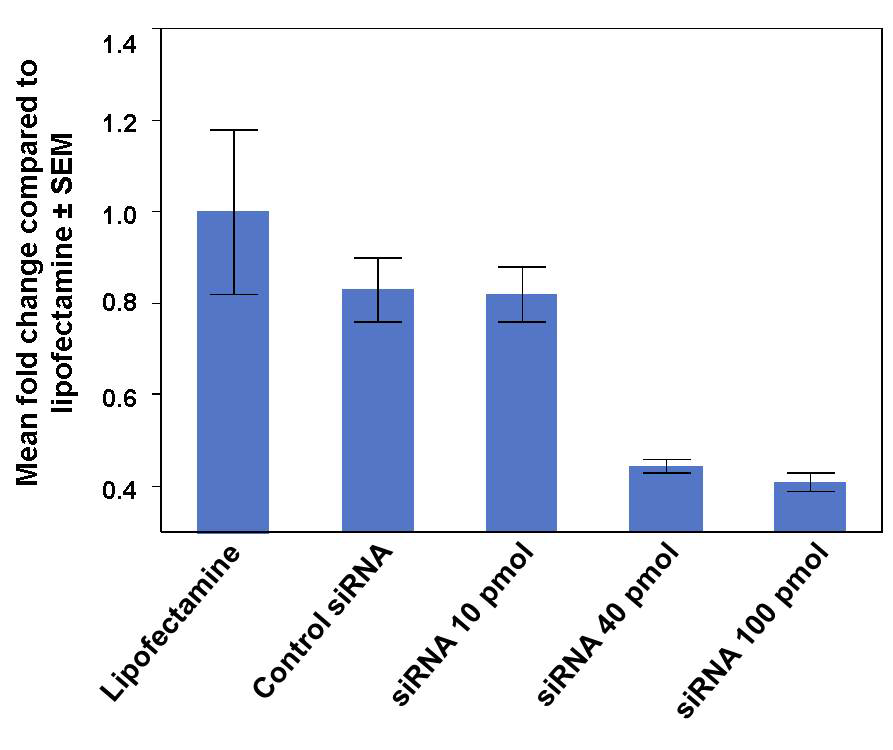

Supplement: Figure S2 — Dose-dependent knock-down of LRRTM3. The fold change of LRRTM3 levels in HEK293T cells are expressed with respect to lipofectamine control. The average normalized Ct values for each experimental group are utilized in the analyses. There were two experiments per group and 4 replicates per experiment. The ∼40% knock-down in LRRTM3 expression was detected for LRRTM3 siRNA SASI_Hs01_00163676 amount of 40 pmols or greater. (TIF) [file pone.0064164.s002.tif]

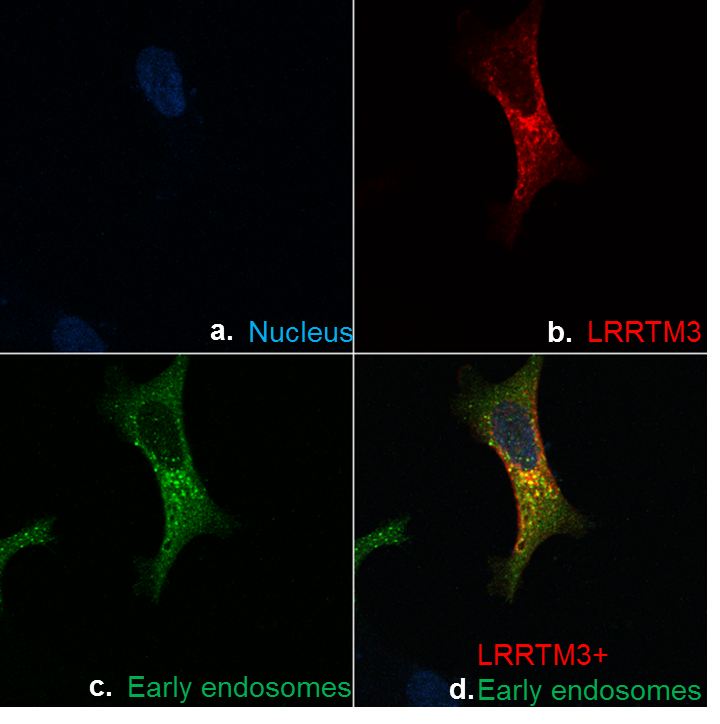

Supplement: Figure S3 — Localization of LRRTM3 and early endosomes (example 2). SH-SY5Y-APP695wt cells were transfected with LRRTM3-V5 and transduced with baculovirus expressing fused early-endosomal protein Rab5a and GFP. Results of staining with a. DAPI (nucleus); b. anti-V5 (LRRTM3); and c. GFP fluorescence indicative of Rab5a expression (early endosomes); d. overlay of a+b+c. There is abundant localization of LRRTM3 within the early endosomes. Magnification:×100. (TIF) [file pone.0064164.s003.tif]

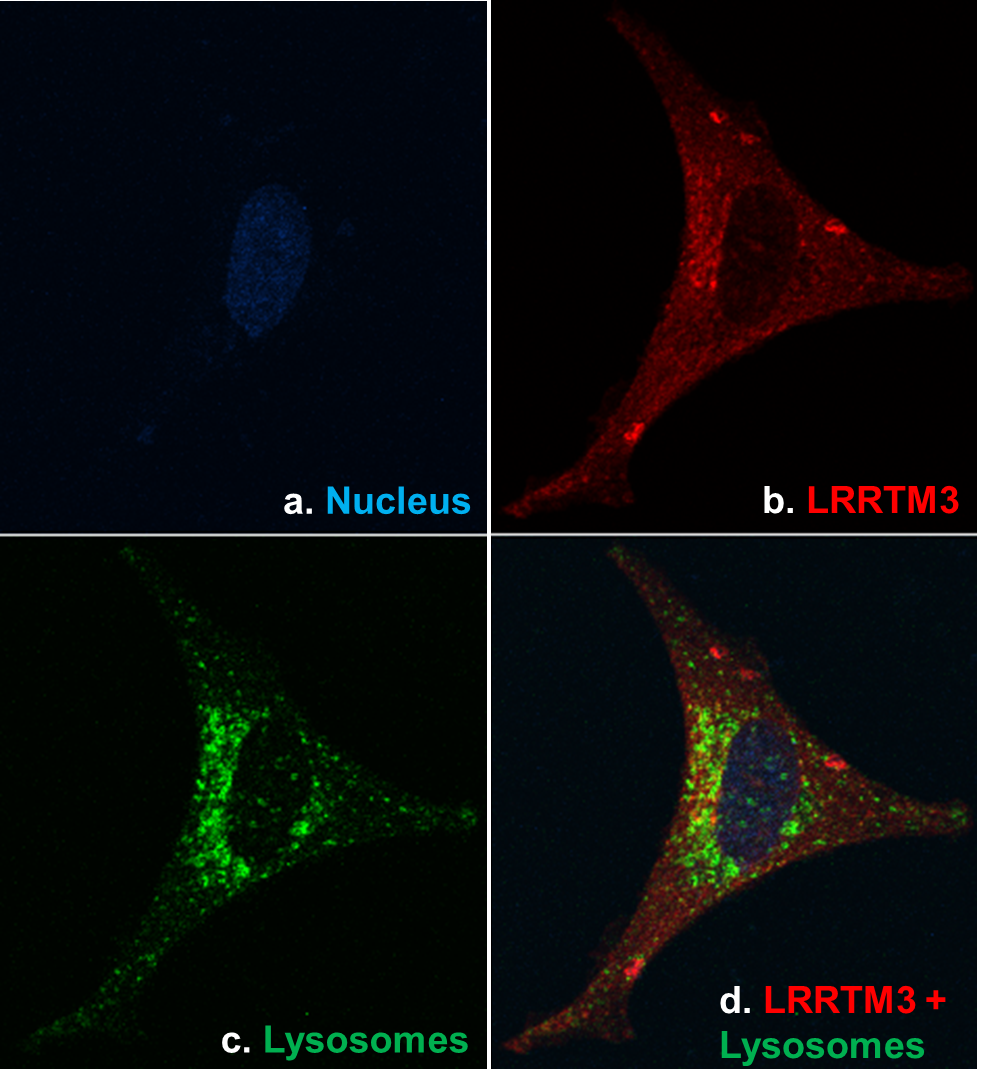

Supplement: Figure S4 — Localization of LRRTM3 and lysosomes. SH-SY5Y-APP695wt cells transfected with LRRTM3-V5 and transduced with baculovirus expressing fused lysosomal protein Lamp1 and GFP. Results of staining with a. DAPI (nucleus); b. anti-V5 (LRRTM3); and c. GFP fluorescence indicative of Lamp1 expression (lysosomes); d. overlay of a+b+c. There does not appear to be localization of LRRTM3 within lysosomes. Magnification: ×100. (TIF) [file pone.0064164.s004.tif]

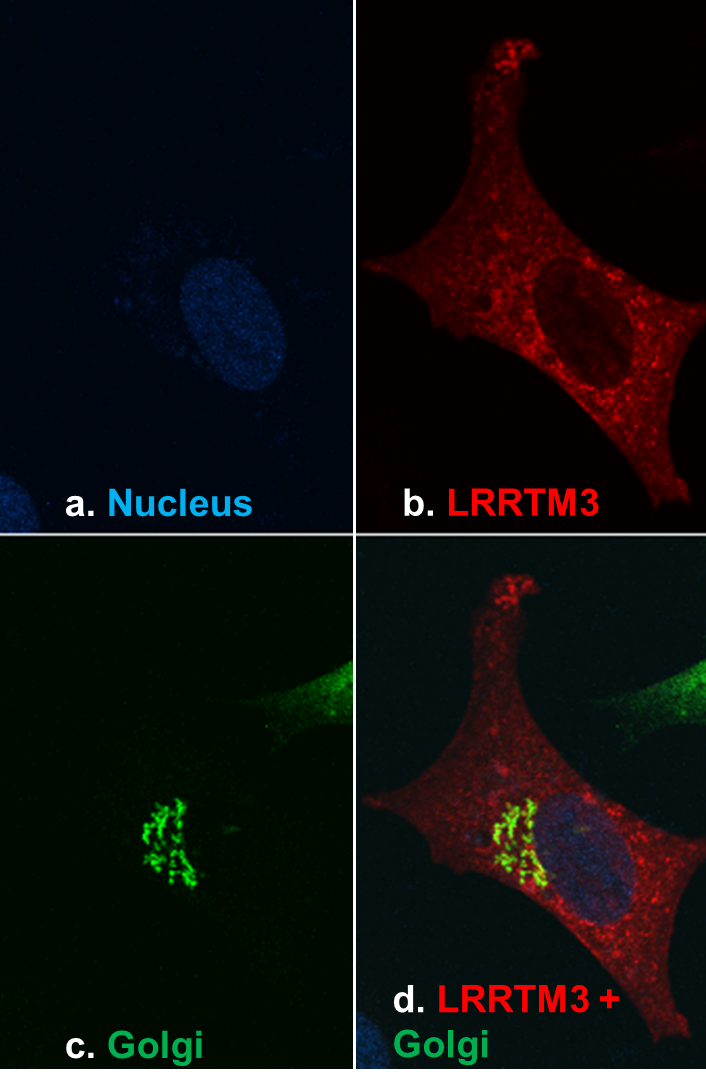

Supplement: Figure S5 — Localization of LRRTM3 and Golgi apparatus. SH-SY5Y-APP695wt cells transfected with LRRTM3-V5 and transduced with baculovirus expressing fused Golgi apparatus protein N-acetylgalactosaminyltransferase 2 and GFP. Results of staining with a. DAPI (nucleus); b. anti-V5 (LRRTM3); and c. GFP fluorescence indicative of N-acetylgalactosaminyltransferase 2 expression (Golgi); d. overlay of a+b+c. There does not appear to be localization of LRRTM3 within the Golgi apparatus. Magnification: ×100. (TIF) [file pone.0064164.s005.tif]

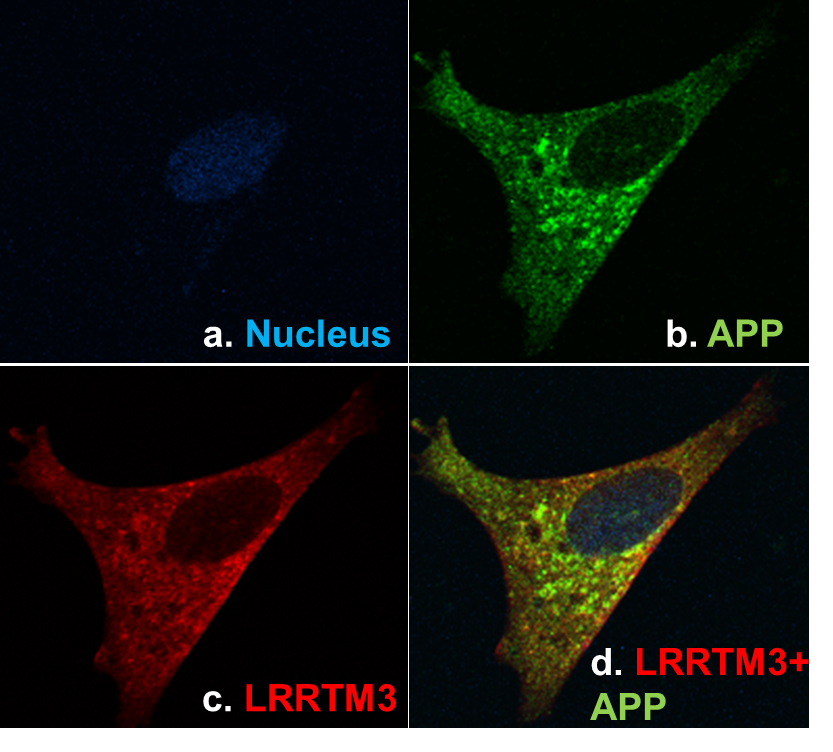

Supplement: Figure S6 — Intracellular co-localization of LRRTM3 and APP (example 2). SH-SY5Y-APP695wt cells were transfected with LRRTM3. Results of staining with a. DAPI (nucleus); b. CT20 (APP); and c. anti-V5 (LRRTM3); d. overlay of a+b+c. There is abundant co-localization of LRRTM3 with APP. Magnification: ×100. (TIF) [file pone.0064164.s006.tif]

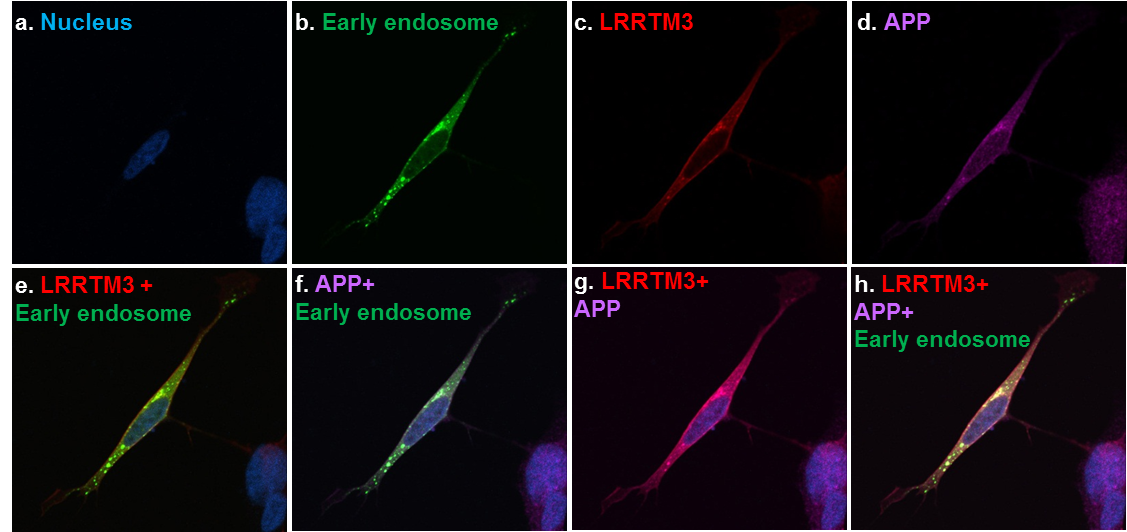

Supplement: Figure S7 — Co-localization of LRRTM3 and APP in early endosomes (example 2). SH-SY5Y-APP695wt cells were transfected with LRRTM3-V5 and transduced with baculovirus expressing fused early-endosomal protein Rab5a and GFP. Results of staining with a. DAPI (nucleus); b. GFP fluorescence indicative of Rab5a expression (early endosomes); c. anti-V5 (LRRTM3); d.CT20 (APP); e. overlay of a+b+c; f. overlay of a+b+d; g. overlay of a+c+d; h.overlay of a+b+c+d. Co-localization of APP, LRRTM3 and early endosomes is visualized as white punctate intracellular structures in h and can also be seen in e-g. The two cells stained for APP and not with LRRTM3 or early endosomes clearly depict a different staining pattern than the cell in the middle of the field, which stains with all three proteins. Magnification: ×63. (TIF) [file pone.0064164.s007.tif]

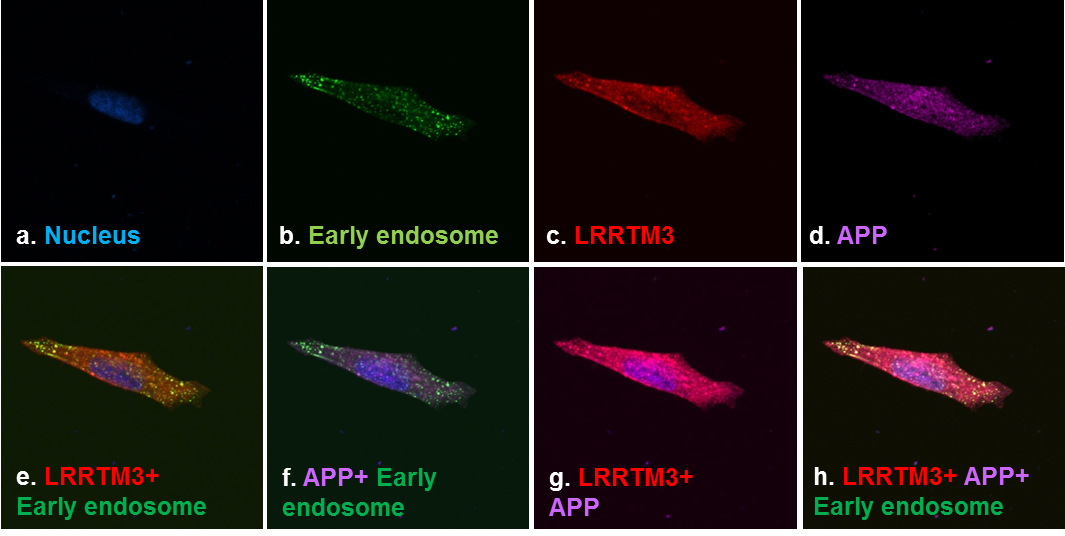

Supplement: Figure S8 — Co-localization of LRRTM3 and APP in early endosomes (example 3). Same conditions and staining are used as Figure S5. (TIF) [file pone.0064164.s008.tif]

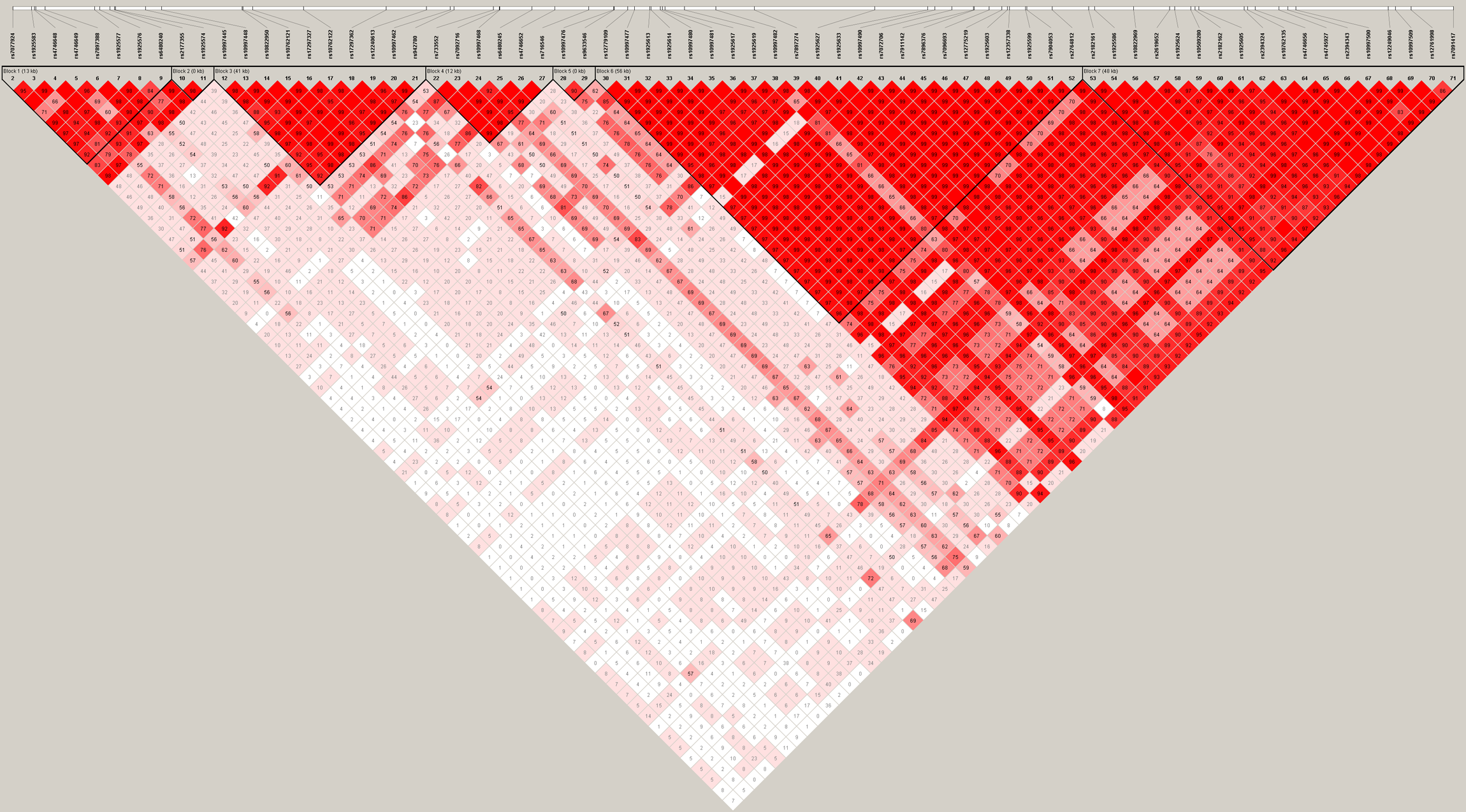

Supplement: Figure S10 — Linkage disequilibrium (LD) plot of 69 SNPs at the LRRTM3 locus. Haploview 4.1 was utilized to obtain the LD plot depicting D’ values for all SNP pairs, using genotypes from Cohort 1. LD blocks are defined according to the solid spine of LD algorithm. SNP names are depicted at the top of the figure. Though, initially 71 SNPs were assessed, two SNPs which violated Hardy-Weinberg equilibrium in controls from either cohort were excluded from the analysis. (TIF) [file pone.0064164.s010.tif]
