# Supplementary material for: LRRTM3 Interacts with APP and BACE1 and Has Variants Associating with Late-Onset Alzheimer’s Disease (LOAD)
Source: PLoS One. 2013 Jun 4;8(6):e64164. doi: 10.1371/journal.pone.0064164 (PMC3672107; doi:10.1371/journal.pone.0064164)
Supplement: Figure S9 — Simple linear regression plots for LRRTM3 vs. other gene brain expression level associations that are significant in Table 1 . Results for cerebellar expression level plots are shown first followed by those for the temporal cortex levels. The p values and correlation coefficients correspond to the plots depicted above them. (DOCX) [file pone.0064164.s009.docx]

**Cerebellum:**

Two sided P < 0.0001

Correlation coefficient (r) = 0.42567 (r² = 0.181195)

Two sided P < 0.0001

Correlation coefficient (r) = 0.525709 (r² = 0.27637)

Two sided P < 0.0001

Correlation coefficient (r) = 0.624343 (r² = 0.389804)

**Temporal Cortex:**

Two sided P = 0.0334

Correlation coefficient (r) = -0.106553 (r² = 0.011354)

Two sided P < 0.0001

Correlation coefficient (r) = 0.591421 (r² = 0.349778)
