# Supplementary material for: LRRTM3 Interacts with APP and BACE1 and Has Variants Associating with Late-Onset Alzheimer’s Disease (LOAD)
Source: PLoS One. 2013 Jun 4;8(6):e64164. doi: 10.1371/journal.pone.0064164 (PMC3672107; doi:10.1371/journal.pone.0064164)
Supplement: Text S1 — Supplementary text. (DOCX) [file pone.0064164.s012.docx]

**LRRTM3 interacts with APP and BACE1 and has variants associating with late-onset Alzheimer’s disease (LOAD).**

Sarah Lincoln, B.Sc.^1#^, Mariet Allen, Ph.D.^1#^, Claire L. Cox^1^, Louise P. Walker^1^, Kimberly Malphrus^1^, Yushi Qiu^1^, Ph.D., Thuy Nguyen^1^, Christopher Rowley^1^, Naomi Kouri^1^, Julia Crook, Ph.D.^2^, V. Shane Pankratz, Ph.D.^3^, Samuel Younkin, Ph.D.^1^, Linda Younkin, Ph.D.^1^, Minerva Carrasquillo, Ph.D.^1^, Fanggeng Zou, Ph.D.^1^, Samer O. Abdul-Hay, Ph.D.^1^, Wolfdieter Springer, Ph.D.^1^, Sigrid B. Sando^4,5^, Jan O. Aasly^4,5^, Maria Barcikowska^6^, Zbigniew Wszolek^7^, Jada M. Lewis, Ph.D.^8^, Dennis Dickson, M.D.^1^, Neill R. Graff-Radford, M.D.^7^, Ronald C. Petersen, M.D., Ph.D.^9^, Elizabeth Eckman, Ph.D.^1,10^, Steven G. Younkin, M.D., Ph.D.^1^, Nilüfer Ertekin-Taner, M.D., Ph.D.^1,7*^

1. Mayo Clinic Florida, Department of Neuroscience, Jacksonville, FL, USA.
2. Mayo Clinic Florida, Biostatistics Unit, Jacksonville, FL, USA.
3. Mayo Clinic Minnesota, Department of Biostatistics, Rochester, MN, USA.
4. Department of Neurology, St.Olav’s Hospital, Edvard Griegs Gate 8, 7006 Trondheim, Norway.
5. Department of Neuroscience, Norwegian University of Science and Technology, NTNU, 7491 Trondheim, Norway.
6. Department of Neurodegenerative Disorders, Medical Research Centre, Polish Academy of Sciences, Warsaw, Poland.
7. Mayo Clinic Florida, Department of Neurology, Jacksonville, FL, USA.
8. Mayo Clinic Minnesota, Department of Neurology, Rochester, MN, USA.
9. MidAtlantic Neonatology Associates, Morristown, NJ, USA (current affiliation).

**Supplementary Methods:**

***LRRTM3 siRNA:***

To test the effect of *LRRTM3* siRNAs, SH-SY5Y cells with stable overexpression of human LRRTM3 with DDK tag (Origene pCMV6 RC211079) were generated. These cells were transfected with pCMV6 vector expressing human full length APP and treated with 100 pmols of three different *LRRTM3* siRNAs (SASI_Hs02_00369484, SASI_Hs01_00163674, SASI_Hs01_00163676) or control siRNA (si-control; MISSION®siRNA) from Sigma Aldrich, using Lipofectamine 2000 (Invitrogen) according to manufacturer’s protocols.

Protein was extracted from harvested cells using RIPA Buffer. After detection of the total protein concentration using Thermo BCA protein assay, equal amounts of protein for each treatment group were separated on a 10% criterion precast gel (Bio-Rad) and electrotransferred to Immobilon P membrane (Millipore, Bedford, MA) at 200 V for 45 minutes. Membranes were blocked in 5% milk TBST and labeled overnight at 4°C with primary antibody (Origene TA50011) against the DDK tag of LRRTM3. Blots were incubated with HRP-linked secondary antibody for 1 hour (Invitrogen), and protein bands were detected using Western Lightning®Plus-ECL (Perkin Elmer). Equivalent sample loading was confirmed by probing the blots with the anti-GAPDH antibody (Pierce/Thermo Scientific). Given its superior knock-down effect, SASI_Hs01_00163676 was chosen for all experiments in this study.

To determine the most effective siRNA dose, we performed a dose-response experiment with SASI_Hs01_00163676. Human embryonic kidney cells (HEK293T) were treated with 10, 40 or 100 pmol of LRRTM3 siRNA, 40 pmol control siRNA or lipofectamine control, followed after 24 hours by harvesting of cells and quantitative PCR (qPCR) using TaqMan assays for human *LRRTM3* (Hs01060657_m1), and *GAPDH* (Hs99999905_m1), *HPRT* (Hs99999909_m1) and *YWHAZ* (Hs00852925_sH) as controls. Each transfection was done twice, and each qPCR experiment was replicated four times. The outliers from the replicates, if present, were excluded until the standard error of the mean (SEM) was ≤0.35. Only those experiments with at least 2 replicates were used in the analyses. The geometric mean of the Cts from three control qPCR assays were obtained for each experiment and subtracted from the average Ct values of the *LRRTM3* assays from the corresponding experiment for normalization. The average normalized Ct values for *LRRTM3* from each experiment was compared against the lipofectamine control to obtain mean fold change.

**LRRTM3 antibodies:**

A number of commercially available antibodies have been tested (HPA024109/Sigma, sc-133384/Santa Cruz Biotechnology, ab106640/ abcam, PA1-757/ Thermo, AF4898/ R&D Systems) by Western blot using human brain, mouse primary neuron protein lysates, and cell lysates from cells overexpressing LRRTM3. These antibodies can detect overexpressed LRRTM3 protein but not endogenous human or mouse LRRTM3.

A polyclonal antibody was also generated by our group using a 21 amino acid peptide (CGVHHELLSHKSFETNAQEDT) near the carboxyl terminus of LRRTM3. The detection of endogenous LRRTM3 with this antibody, likewise, is not robust.

**Supplementary Results:**

***LRRTM3 siRNA:***

Three siRNAs against LRRTM3 were tested and they all led to knock-down of this gene (**Supplementary Figure S1**) in SH-SY5Y cells with stable overexpression of LRRTM3-DDK, although SASI_Hs01_00163676 had the strongest knock-down effect. The dose-response experiments conducted in HEK293T cells revealed LRRTM3 siRNA SASI_Hs01_00163676 amount ≥40 pmols to lead to effective knock-down of ~40% compared to lipofectamine control (**Supplementary Figure S2**). Thus 50 pmols of *LRRTM3* siRNA SASI_Hs01_00163676 was used in all downstream experiments.
